# Synthesis, Reactivity and Structural Properties of Trifluoromethylphosphoranides

**DOI:** 10.1002/chem.202104308

**Published:** 2022-02-09

**Authors:** Oleg O. Shyshkov, Alexander A. Kolomeitsev, Berthold Hoge, Enno Lork, Axel Haupt, Mira Keßler, Gerd‐Volker Röschenthaler

**Affiliations:** ^1^ Department of Life Sciences and Chemistry Jacobs University Bremen gGmbH Campus Ring 1 28759 Bremen Germany; ^2^ Faculty 2: Biology/Chemistry Institute of Inorganic Chemistry and Crystallography Universität Bremen Leobener Straße 7 28359 Bremen Germany; ^3^ Centrum für Molekulare Materialien Fakultät für Chemie Universität Bielefeld Universitätsstraße 25 33615 Bielefeld Germany

**Keywords:** fluorine, fluorination, hypervalent compounds, phosphorus, trifluoromethylation

## Abstract

Phosphoranides are interesting hypervalent species which serve as model compounds for intermediates or transition states in nucleophilic substitution reactions at trivalent phosphorus substrates. Herein, the syntheses and properties of stable trifluoromethylphosphoranide salts are reported. [K(18‐crown‐6)][P(CF_3_)_4_], [K(18‐crown‐6)][P(CF_3_)_3_F], and [NMe_4_][P(CF_3_)_2_F_2_] were obtained by treatment of trivalent precursors with sources of CF_3_^−^ or F^−^ units. These [P(CF_3_)_4‐n_F_n_]^−^ (n=0–2) salts exhibit fluorinating (n=1–2) or trifluoromethylating (n=0) properties, which is disclosed by studying their reactivity towards selected electrophiles. The solid‐state structures of [K(18‐crown‐6)][P(CF_3_)_4_] and [K(18‐crown‐6)][P(CF_3_)_3_F] are ascertained by single crystal X‐ray crystallography. The dynamics of these compounds are investigated by variable temperature NMR spectroscopy.

## Graphical abstract

**Different trifluoromethylated phosphoranides** are accessible upon treatment of trifluoromethylated phosphanes, such as P(CF_3_)_3_, with a source of F^−^ or CF_3_^−^ in presence of a stabilizing counterion. The hypervalent compounds have been investigated by NMR spectroscopic as well as X‐ray crystallographic methods, gaining insights into their stability and structural behavior. In addition, the phosphoranides have revealed fluorinating and trifluoromethylating properties towards electrophiles.

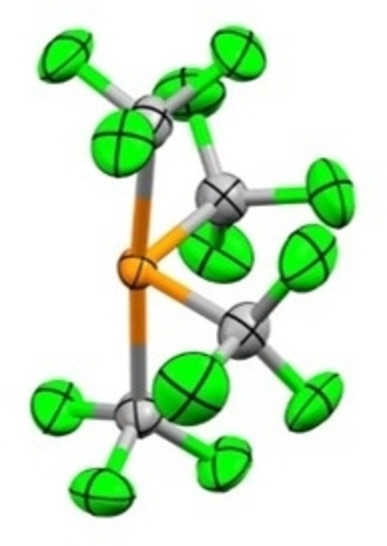

## Introduction

To date, a number of hypervalent phosphorus compounds has been studied, including phosphoranides which feature a formally negatively charged, tetracoordinated phosphorus atom. They turned out to be useful models for the intermediates or transition states in nucleophilic substitution reactions at trivalent phosphorus substrates.^[1]^ In general, phosphoranides are accessible by three major approaches, namely the addition of X^−^ to a phosphorus(III) compound (Lewis acid‐base interaction),^[2]^ deprotonation of a pentavalent phosphorane R_4_PH,^[3]^ or oxidation of an anionic and monovalent species, such as [P(CN)_2_]^−^ by Cl_2_ or Br_2_.^[4]^ As a rule of thumb, phosphoranides have to be stabilized by electron‐withdrawing substituents X with a low tendency to function as leaving groups at the same time. The first phosphoranide to be isolated was [PBr_4_]^−^, followed by the chloro‐ and fluoro‐analogues [PCl_4_]^−^ and [PF_4_]^−^.[^2^, ^5^, ^6^] However, tetraiodo phosphoranide [PI_4_]^−^ has eluded observation so far, which is in line with the decreasing acceptor properties of the phosphanes within the homologous series. In solution, the equilibrium between PX_3_, X^−^ and [PX_4_]^−^ lies well on side of the educts for X=Br and to some extent on side of the educts for X=Cl, whereas it lies on side of the phosphoranide for X=F.[^2^, ^5^, ^6^] Structurally, the phosphoranides are derived from a trigonal bipyramid, with the sterically active lone‐pair in an equatorial position. Axial and equatorial substituents of the [PF_4_]^−^ ion are interconverted via Berry pseudo rotation resulting in indifferent NMR resonances at room temperature. However, the exchange is slowed down at low temperatures, facilitating the differentiation of two types of substituents via NMR spectroscopy.^[6]^ Apart from the aforementioned tetrahalophosphoranides, a few cyclic organophosphoranides have also been described.[^3^, ^7^] Moreover, a few derivatives have been prepared containing both, organic residues and (pseudo‐)halide groups at the phosphorus atom, for example [PR(CN)_2_X]^−^ with R=Me, Et, Ph, C_6_F_5_ and X=Cl, Br, I.^[8]^ Basically, halide substituents at [PX_4_]^−^ may be replaced by perfluoroalkyl functionalities. Thus, trifluoromethylated phosphoranides had been predicted to be relatively stable, long before their verification by experiment,^[1]^ and the analogous pentafluoroethylated phosphoranides have been reported only recently.^[9]^ The homoleptic tetrakis(trifluoromethyl)phosphoranide has been authenticated as an anion in the extremely unstable salt [(Me_2_N)_3_S][P(CF_3_)_4_] and in a slightly more stable, but nevertheless still very reactive and pyrophoric, tetramethylammonium derivative, [NMe_4_][P(CF_3_)_4_].^[10]^ A similar increase in stability has been observed for the corresponding salts [(Me_2_N)_3_S][P(CF_3_)_3_F] and [NMe_4_][P(CF_3_)_3_F]. The difluorobis(trifluoromethyl)‐phosphoranide [P(CF_3_)_2_F_2_]^−^ results from difluorocarbene extrusion of [P(CF_3_)_3_F]^−^ or by addition of NMe_4_F to the phosphine P(CF_3_)_2_F.^[11]^ So far, detailed NMR‐spectroscopic and crystallographic examinations of these compounds have been thwarted by their instability. The most suitable starting material for the preparation of trifluoromethyl‐containing phosphoranides is P(CF_3_)_3_, which is well known as a ligand in transition metal coordination chemistry.^[12]^ This phosphine may be prepared for example by treatment of CF_3_I with white phosphorus, by reaction of Cd(CF_3_)_2_ with PI_3_, by reduction of the phosphorane (CF_3_)_3_PF_2_, or by combining CF_3_Br with P(NEt_2_)_3_ and P(OPh)_3_.^[13]^ The aforementioned methods, however, all have significant drawbacks, since they are either inconvenient, cost‐intensive or require the employment of toxic or environmentally harmful compounds. Thus, an alternate synthesis involving relatively non‐hazardous, commercially available reagents was developed. The reaction of triphenyl phosphite with Me_3_SiCF_3_ in the presence of an equimolar amount of CsF afforded P(CF_3_)_3_ in 98 % yield. Using only catalytic amounts of CsF, at least 90 % yield were accomplished.^[14]^

Herein, we would like to report upon the synthesis, properties, and reactivity of stable trifluoromethyl phosphoranides derived from P(CF_3_)_3_.

## Results and Discussion

**Scheme 1:**
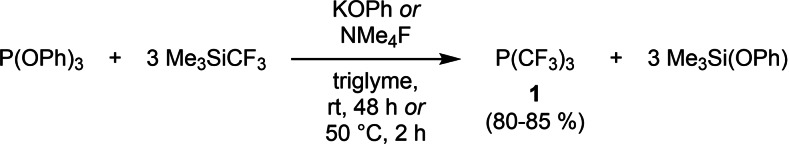
Preparation of P(CF_3_)_3_ (**1**). Triglyme=2,5,8,11‐tetraoxadodecane.

P(CF_3_)_3_ (**1**) was obtained by treatment of P(OPh)_3_ with Me_3_SiCF_3_ in the presence of small amounts of NMe_4_F or KOPh in ethereal solvents, after a modified literature procedure. ^[14]^ The reaction is performed in the temperature range 20 to 50 °C, affording phosphine **1** in yields of 80–85 % (Scheme 1).^[15]^ The starting materials are preferably used stoichiometrically to avoid contamination with by‐products. An excess of the phosphite leads to the formation of (CF_3_)P(OPh)_2_ and CF_3_H, whereas larger amounts of the silane mainly produce traces of CF_3_H. The product is easily pumped off and subsequently distilled at atmospheric pressure.

**Scheme 2:**
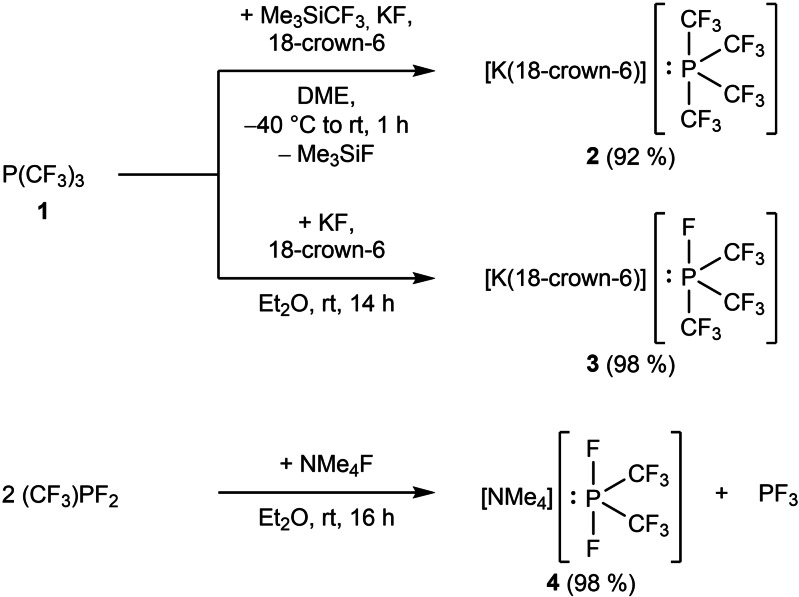
Preparation of stable trifluoromethylated phosphoranides **2**, **3** and **4**.

Treatment of P(CF_3_)_3_ (**1**) with Me_3_SiCF_3_ in the presence of fluoride salts leads to the formation of corresponding [P(CF_3_)_4_]^−^ salts. To obstruct the notorious tendency to decompose, the [NMe_4_]^+^ ion in [NMe_4_][P(CF_3_)_4_]^[10]^ should be replaced by more bulky cations, such as [K(18‐crown‐6)]^+^ which previously allowed synthesis and X‐ray structural characterization of [K(18‐crown‐6)][P(CF_3_)_2_].^[16]^ Therefore, we attempted the preparation of stable phosphoranides [P(CF_3_)_3_F]^−^ and [P(CF_3_)_4_]^−^ by combination of P(CF_3_)_3_ with KF/18‐crown‐6 or KF/Me_3_SiCF_3_/18‐crown‐6, respectively. Compounds [K(18‐crown‐6)][P(CF_3_)_4_] (**2**) and [K(18‐crown‐6)][P(CF_3_)_3_F] (**3**) are accessible by this approach in 92 % and 98 % yield (Scheme 2).^[15]^ Attempts to prepare pure salts of the difluorobis(trifluoromethyl)phosphoranide ion, [P(CF_3_)_2_F_2_]^−^, failed when P(CF_3_)_2_F was reacted with NMe_4_F or KF/18‐crown‐6. With large fluoride excess, a maximum of 60 % phosphoranide was achieved. In a consecutive reaction of [P(CF_3_)_2_F_2_]^−^ with the starting phosphine, [P(CF_3_)_2_F_3_{P(CF_3_)_2_}]^−^ was formed as a byproduct. The reaction is believed to proceed via an adduct formed by the phosphoranide and phosphine, [P(CF_3_)_2_F{P(CF_3_)_2_F_2_}]^−^, and subsequent fluoride transfer.

Interestingly, [NMe_4_][P(CF_3_)_2_F_2_] (**4**) is formed, when the mono‐trifluoromethyl phosphine (CF_3_)PF_2_ is allowed to react with NMe_4_F in diethyl ether at room temperature (Scheme 2).^[15]^ As a side product, PF_3_ is liberated, which implies a low affinity between PF_3_ and CF_3_^−^ and therefore rationalizes the preferred formation of [P(CF_3_)_2_F_2_]^−^ instead of expected [P(CF_3_)F_3_]^−^. The latter mono‐trifluoromethylated species is still unknown and all our attempts to synthesize and detect this species failed. Thus, combination of (CF_3_)PF_2_ with NMe_4_F in CH_3_CN or treatment of PF_3_ with NMe_4_F and Me_3_SiCF_3_ did not give unambiguous evidence for the transient existence of [NMe_4_][P(CF_3_)F_3_].

Next, we looked at the stability of the novel trifluoromethylphosphoranide salts **2** and **3** in comparison to the related [NMe_4_]^+^ compounds. Disregarding the nature of the counter ion, most of the trifluoromethylated phosphoranides suffer from facial difluorocarbene elimination, leading to a mixture of (trifluoromethyl)fluoro derivatives.

The facile loss of a CF_3_ group may be due to the axial 3‐center‐4‐electron bonding. Consistently, apical P−C bonds are weakened in comparison to 2‐center‐2‐electron P−C bonds. In stark contrast to the corresponding [NMe_4_]^+^ salts, the decomposition of which commences at about −45 °C in dimethoxyethane (DME) solution, derivative [K(18‐crown‐6)][P(CF_3_)_4_] (**2**) decomposes very slowly at ambient temperature. The stability of **2** is considerably improved in the presence of an equivalent amount of P(CF_3_)_3_ (**1**). Thus, 74 % of **2** remained intact after 24 h in DME solution mixed with **1**. A similar behavior was encountered for DME solutions of [K(18‐crown‐6)][P(CF_3_)_3_F] (**3**) and the analogous [NMe_4_][P(CF_3_)_3_F]. [NMe_4_][P(CF_3_)_3_F] decomposed in DME solution at room temperature within two weeks, whereas 85 % of **3** remain unaffected under these conditions. In addition, storage of solid [NMe_4_][P(CF_3_)_3_F] at 20 °C for 12 days led to a 2 : 1 mixture of [NMe_4_][P(CF_3_)_3_F] and [NMe_4_][P(CF_3_)_2_F_2_].

Solid [NMe_4_][P(CF_3_)_4_] tends to spontaneously explode, whereas solid [K(18‐crown‐6)][P(CF_3_)_4_] (**2**) is relatively stable at room temperature. The remarkable instability of the ammonium salt may be rationalized by the reaction of the strong base CF_3_^−^ with [NMe_4_]^+^ which would result in the formation of the very reactive and unstable ammonium ylide Me_3_N^+^−CH_2_^−^.^[17]^ Analysis of the decomposition products revealed only traces of P(CF_3_)_3_, but significant amounts of CF_3_H and NMe_3_. We also found, that :CF_2_ preferentially inserts into α‐C−H bonds of ethereal solvents, as it has been described earlier.^[18]^

Clearly, all trifluoromethyl phosphoranides under discussion are thermally unstable and very reactive. The obtained products are colorless to pale yellow solids which are sensitive towards moisture and oxygen, the latter causing spontaneous ignition. Analytically pure compounds are only obtained by drying *in vacuo* at 0 to −30 °C. In contrast, drying at room temperature leads invariantly to impure samples. Moreover, the phosphoranides are not characterized by sharp melting points, since they always suffer from decomposition prior to liquefaction. Thereby, the [NMe_4_]^+^ salts decompose and melt at lower temperatures than the corresponding compounds **2** and **3**, featuring cation [K(18‐crown‐6)]^+^. Pyrolysis of [NMe_4_]^+^ phosphoranides yields mixtures of PF_3_, P(CF_3_)_3_, CF_3_H and NMe_3_ in addition to an intractable black solid, whereas [K(18‐crown‐6)]^+^ salts mainly liberate P(CF_3_)_3_ as well as some PF_3_ and CF_3_H. Fast hydrolysis of the phosphoranides in wet DME yields [PH(CF_3_)(=O)O]^−^, whereby [P(CF_3_)_3_F]^−^ is slightly less readily hydrolyzed than [P(CF_3_)_4_]^−^. The hydrolysis of [PH(CF_3_)(=O)O]^−^ is very slow, and requires a few drops of aqueous NaOH to produce CF_3_H and HP(=O)(OH)_2_.

The remarkable reactivity of the phosphoranides under discussion is based upon the loss of an axial ligand which may be conveniently trapped by suitable electrophiles. In keeping with this, [P(CF_3_)_4_]^−^ is a powerful trifluoromethylating reagent, whereas the anions [P(CF_3_)_3_F]^−^ and [P(CF_3_)_2_F_2_]^−^ behave as fluoride donors. This is nicely illustrated by the clean reaction of **2**, **3**, and **4** with Me_3_SiCl which furnished products Me_3_SiCF_3_ and Me_3_SiF, respectively.

**Scheme 3:**
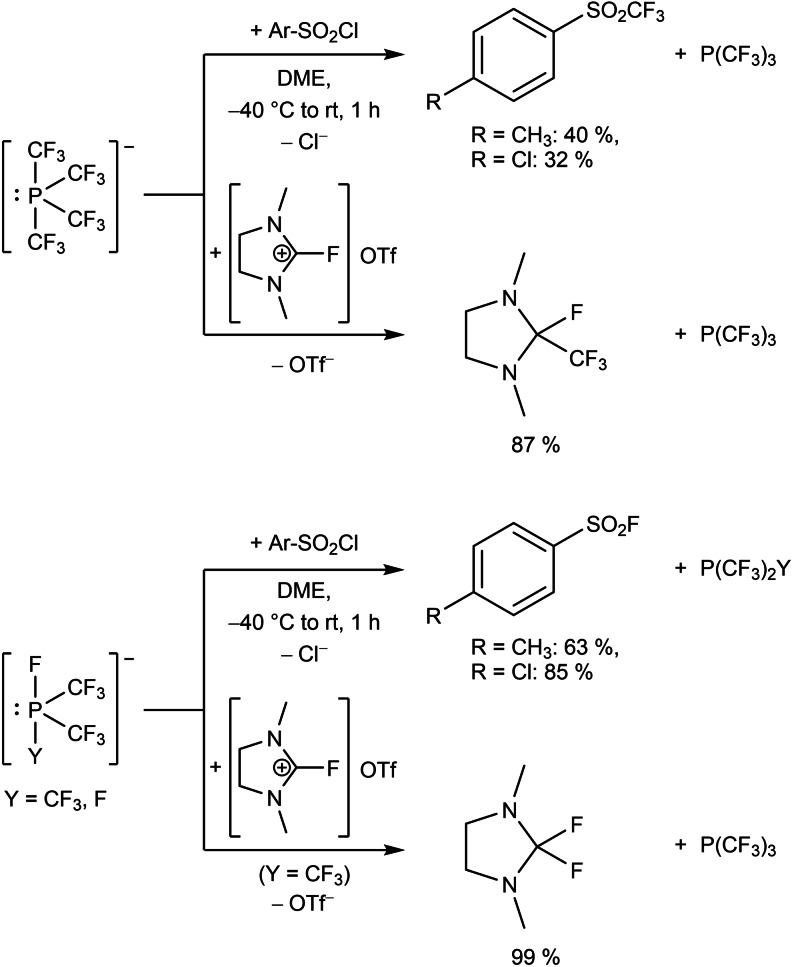
Fluorination and trifluoromethylation by trifluoromethylated phosphoranides.

The ammonium salt [NMe_4_][P(CF_3_)_2_ClF] is generated in quantitative yield, if [NMe_4_][P(CF_3_)_2_F_2_] is added to an equimolar amount of Me_3_SiCl at −30 °C. On the other hand, reactions of the phosphoranides [P(CF_3_)_2_F_2_]^−^ and [P(CF_3_)_3_F]^−^ with Me_3_SiCF_3_ yield [P(CF_3_)_4_]^−^. Upon warming of the reaction mixture from −60 °C to room temperature, the phosphoranides also react with SO_2_ and aryl sulfonyl chlorides Ar−SO_2_Cl under formation of [X‐SO_2_]^−^ and Ar−SO_2_X, respectively (Scheme 3).^[15]^ Moreover, boric esters and aldehydes are trifluoromethylated by [P(CF_3_)_4_]^−^ with CF_3_H as a major side product. Thus, in the reaction of **2** with B(OMe)_3_, [K(18‐crown‐6)][B(OMe)_3_(CF_3_)] is formed. Treatment of **2** with benzaldehyde and subsequent aqueous workup yields PhCH(CF_3_)(OH).

Interestingly, trifluoromethyl phosphoranides do not react with neutral 1,3‐dimethyl‐2,2‐difluoroimidazolidine (DFI). However, the corresponding imidazolium triflate is trifluoromethylated by [P(CF_3_)_4_]^−^ or fluorinated by [P(CF_3_)_3_F]^−^ under liberation of P(CF_3_)_3_ (Scheme 3).

Furthermore, trifluoromethylated phosphoranides are prone to oxidation. Thus, high yields of sixfold coordinated trifluoromethyl phosphates [P(CF_3_)_3_XY_2_]^−^ (X=CF_3_, F; Y=Cl; F) are generated upon oxidation of the phosphoranides with Cl_2_ or Deoxo‐Fluor® ((MeOCH_2_)_2_NSF_3_) at low temperature. However, treatment of the phosphoranides with hexafluoroacetone does not lead to the oxidation of the phosphorus center. Transfer of CF_3_^−^ or F^−^ anions to the ketone under formation of [(F_3_C)_3_CO]^−^ or [F(F_3_C)_2_CO]^−^ was observed, instead.

**Scheme 4:**
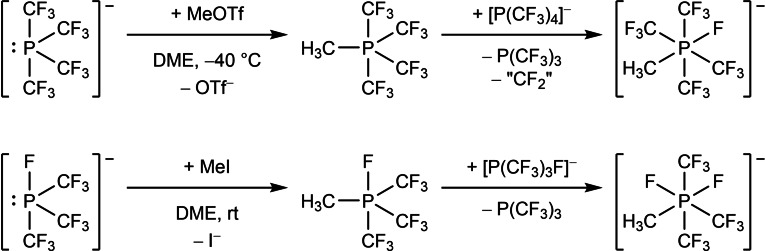
Methylation of trifluoromethylated phosphoranides and subsequent reaction with a second equivalent phosphoranide.

Methylation of the phosphoranides yields pentavalent trifluoromethyl phosphates (Scheme 4). In case of [P(CF_3_)_4_]^−^, methyl transfer is achieved by MeOTf in DME at −40 °C. For the methylation of [P(CF_3_)_3_F]^−^, MeI has been used in DME solution at room temperature.^[15]^ Since the initially formed phosphoranes are strong Lewis acids, they cannot be isolated but immediately abstract CF_3_^−^ or F^−^ from a second equivalent of the starting phosphoranide. Here should be stated that the conceivable addition of the CF_3_^−^ anion on a phosphorane is limited by steric hindrance, and fluoride addition to the phosphorane is preferred. Thus, methylation of [P(CF_3_)_4_]^−^ yields [P(CH_3_)(CF_3_)_4_F]^−^ and methylation of [P(CF_3_)_3_F]^−^ yields [P(CH_3_)(CF_3_)_3_F_2_]^−^.

**Figure 1:**
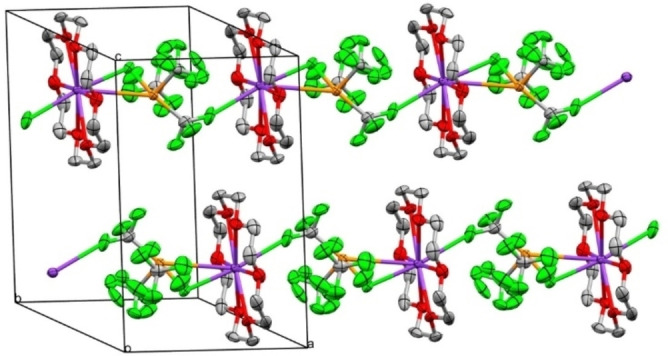
Solid‐state packing of [K(18‐crown‐6)][P(CF_3_)_3_F] (3) exhibiting zig‐zag chains along the a‐axis. The equatorial CF_3_ groups are disordered. Ellipsoids are at a probability level of 50 %, hydrogen atoms are omitted for clarity.

**Figure 2:**
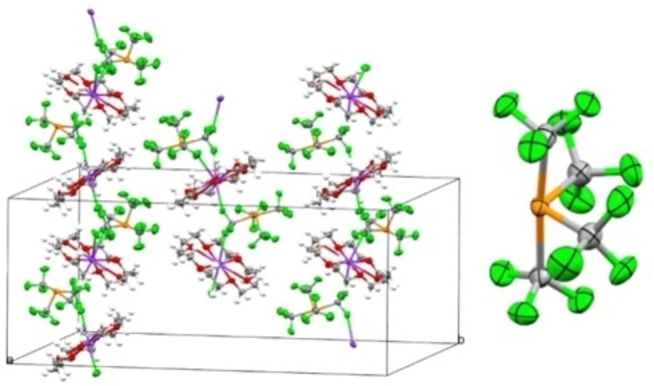
Extract of the solid‐state packing of [K(18‐crown‐6)][P(CF_3_)_4_] (**2**) revealing zig‐zag chains along the c‐axis (left) and structure of a [P(CF_3_)_4_]^−^ anion (right). Ellipsoids are at a probability level of 50 %, hydrogen atoms are omitted for clarity.

Elucidation of the solid‐state structures of the compounds [K(18‐crown‐6)][P(CF_3_)_3_F] (**3**) (Figure 1) and [K(18‐crown‐6)][P(CF_3_)_4_] (**2**) (Figure 2) were effected by means of single crystal X‐ray crystallography.^[19]^ [P(CF_3_)_3_F]^−^ salt **3** crystallizes in the triclinic space group *P‐1*, whereas [P(CF_3_)_4_]^−^ salt **2** crystallizes in the monoclinic space group *P2_1_/c* with three geometrically nonequivalent anions, therefore average values of structural parameters of **2** are given hereinafter. In both cases, the coordination geometry of the phosphoranide anions is based on distorted trigonal bipyramids, where the lone pair at the phosphorus center is sterically active and occupies one of the equatorial positions. For both compounds, a few structural similarities are observed. The axial bonds in the phosphoranides (**3**: d(P‐CF_3ax_)=1.97(2) Å, d(P−F)=1.79(1) Å; **2**: d(P‐CF_3ax_)=2.049 Å) are elongated with respect to the neutral species PF_3_ (d(P−F)=1.570(1) Å, gas phase electron diffraction – GED)^[20]^ and P(CF_3_)_3_ (**1**) (d(P‐CF_3_)=1.93(2) Å, GED)^[21]^ due to the 3‐center‐4‐electron bonding. The axial P−F bond is significantly shorter than the axial P−C bond in the [P(CF_3_)_3_F]^−^ anion of compound **3**, as could be expected. The equatorial P−CF_3_ bonds of **2** are 1.894 Å on average and thus, comparable to the P−C bonds in the neutral compounds P(CF_3_)_3_ (**1**) (1.93(2) Å, GED)^[21]^ or (F_3_C)_2_P−P(CF_3_)_2_ (1.886(4) and 1.880(4) Å via X‐Ray; 1.90 Å via GED).^[22]^

Furthermore, the CPC‐angles between the two equatorial CF_3_‐groups are almost identical in **2** and **3** (104.06(2) to 105.05(1)°), but slightly larger than the corresponding CPC‐angle in the neutral phosphine P(CF_3_)_3_ (**1**) (100(3)°, GED). The angles between the apical substituents are smaller than 180° and amount to 170.47(1)° for **3** and 174.74(1) to 176.02(1)° for **2**. The reasons for this distortion are repulsive interactions of the axial substituents with the lone pair, which is especially pronounced for the apical fluorine substituent in compound **3**. Thus, the CPC angles between axial and equatorial CF_3_‐groups in both anions are virtually identical (90.79(1)° and 89.94(1)° in **3** and on average, 89.1° in **2**).

The phosphoranide salts **3** and **2** differ in their solid‐state packing, since [P(CF_3_)_4_]^−^ in **2** forms zig‐zag chains along the c‐axis. The chains are composed of K^+^ centers which are symmetrically surrounded by 18‐crown‐6 and two phosphoranide anions. The bridging between two K‐centers is realized by two fluorine atoms of each a single axial CF_3_ group of the phosphoranide. In each cell, there are three geometrically inequivalent anions. In contrast, [P(CF_3_)_3_F]^−^ in compound **3** has two ion‐pairs per unit cell and exhibits a comparably simple arrangement, resulting in zig‐zag chains along the a‐axis of the unit cell. Here, bridging between the cations is realized by the two axial substituents of the phosphoranide anion, i. e. the axial fluorine atom and one of the fluorine atoms of the axial CF_3_ group. For both phosphoranide salts **2** and **3**, the geometry of the cations and anions is determined by the pursuit for a maximum number of K−F contacts with preferably minimal distances. Since one observes rather large K−P distances of 4.454(3) to 5.436(7) Å (**2**) and 3.649(9) Å (**3**), respectively, this indicates in both cases a packing of well‐isolated ions.

**Figure 3:**
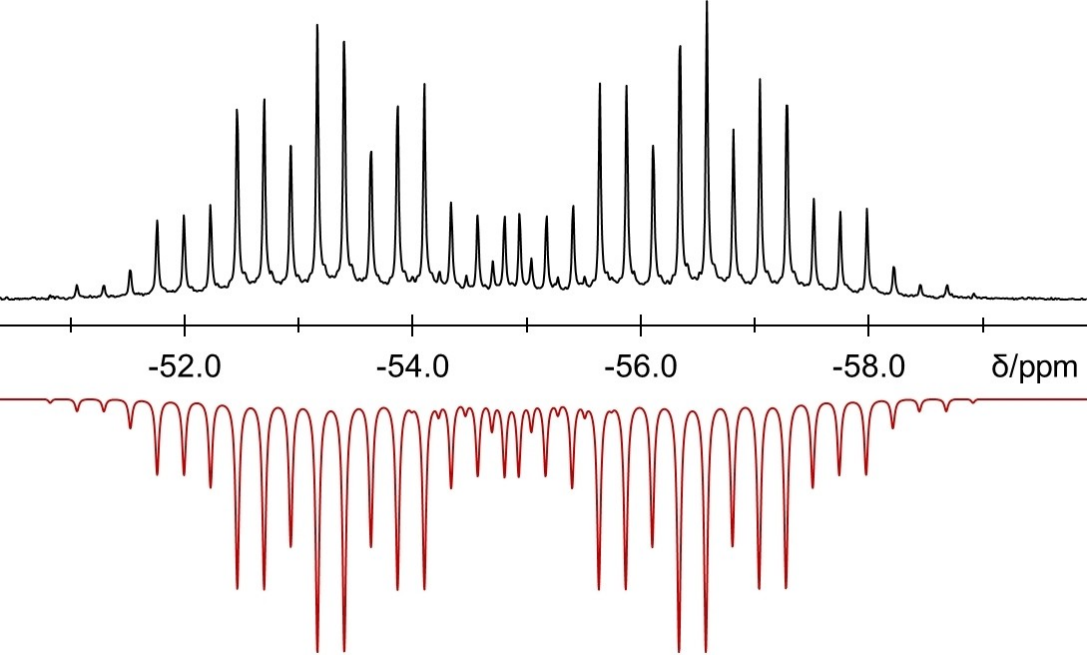
Experimental (top) and simulated^[23]^ (bottom) ^31^P NMR resonance of [NMe_4_][P(CF_3_)_3_F] at −50 °C. The splitting may be described as a doublet of septets of quartets. Simulation yields the coupling constants ^*1*^ *J*(P−F)=385 Hz; ^*2*^ *J*(P‐C**F**_3eq._)=85 Hz and ^*2*^ *J*(P‐C**F**_3ax._)=29 Hz.

Furthermore, trifluoromethyl phosphoranides **2** and **3** were studied by variable temperature NMR spectroscopy. Their spectra are compared with those of the corresponding tetramethylammonium derivatives. Phosphoranides may undergo inter‐ and intramolecular exchange processes. They tend to form an equilibrium with their precursors in solution, and pseudorotation may lead to an exchange of equatorial and axial substituents. In keeping with this, the ^31^P NMR spectrum of [NMe_4_][P(CF_3_)_3_F] shows a slightly broadened decet at −38 °C in acetonitrile solution, whereas the ^19^F NMR spectrum is characterized by a broad singlet and a doublet in the ratio 1 : 9. The lack of fine coupling implies a fast exchange process. Upon addition of excess NMe_4_F this exchange is considerably slowed down, so that a doublet of quartets of septets can be distinguished in the ^31^P NMR spectrum. In contrast, the exchange is very slow in DME solution, which allows differentiation of the axial and equatorial CF_3_ groups. However, a slow exchange remains as evidenced by slightly broad signals. Again, these dynamics can be suppressed by addition of small amounts NMe_4_F resulting in a well resolved ^31^P NMR resonance at room temperature. Cooling a DME solution of [NMe_4_][P(CF_3_)_3_F] to −60 °C in absence of NMe_4_F leads to the same observation. A fast exchange process takes place in DME above 60 °C, which can be identified by variable temperature ^31^P NMR spectroscopy. At −50 °C the ^31^P NMR signal is fully resolved (Figure 3), whereas a broad multiplet is found at about 50–60 °C, finally resulting in a broad decet at 80 °C. Consequently, the CF_3_ groups give rise to two broad singlets in the ^19^F NMR spectrum at 50 °C which fuse to one broad singlet at 60 °C.

In contrast, ^19^F and ^31^P NMR spectra of [K(18‐crown‐6)][P(CF_3_)_3_F] (**3**) in DME at −90 °C do not allow a differentiation between axial and equatorial CF_3_ groups. The appearance of the spectra points to a fast exchange process similar to that observed in MeCN solution. We investigated the influence of the counterions on the spectra in DME in greater detail. If a mixture of KF/18‐crown‐6 is added to a DME solution of [NMe_4_][P(CF_3_)_3_F], no changes in the NMR resonances take place. In contrast, the addition of NMe_4_F to [K(18‐crown‐6)][P(CF_3_)_3_F] (**3**) results in dramatic transformations in the spectrum. Exchange processes are significantly slowed down, so that partially resolved signals are observable in the same way as for [NMe_4_][P(CF_3_)_3_F] in MeCN at −60 °C.

Presumably, [NMe_4_][P(CF_3_)_3_F] exists as a tight pair of contact ions in DME, which leads to decelerated exchange processes. The [NMe_4_]^+^ cation probably interacts with the phosphorus atom, leading to the formation of P−F bonds. On the other hand, the [K(18‐crown‐6)]^+^ cation rather interacts with the axial fluorine atom of the anion than with the phosphorus atom, as evident from the molecular structure, facilitating a fast exchange process.

The resonances of the axial and equatorial CF_3_ groups of [K(18‐crown‐6)][P(CF_3_)_4_] (**2**) are fully resolved at −50 °C. Upon warming an intermediate behavior is observed at 0 °C, whereas a fast exchange process occurs at 80 °C. In comparison, [NMe_4_][P(CF_3_)_4_] exhibits fully resolved spectra at −45 °C but it cannot be observed above 10 °C due to its thermal instability.

In solution, [P(CF_3_)_4_]^−^ and [P(CF_3_)_3_F]^−^ are generally stabilized by the addition of P(CF_3_)_3_ (**1**). Since phosphoranides and their precursors form an equilibrium in solution, the addition of one of the reactants entails a shift of this equilibrium to the side of the phosphoranide. Thus, [K(18‐crown‐6)][P(CF_3_)_4_] (**2**) already exhibits resolved ^19^F NMR spectra with fine splitting at −30 °C in the presence of P(CF_3_)_3_ (**1**). The NMR resonances of [K(18‐crown‐6)][P(CF_3_)_3_F] (**3**), on the other hand, are broadened, which might arise from interactions of the axial fluorine substituent of the phosphoranide with the phosphine.

## Conclusion

In conclusion, we have demonstrated that stable trifluoromethylated phosphoranides are accessible upon treatment of trifluoromethyl phosphines with a source of F^−^ or CF_3_^−^, respectively. The anionic species are efficiently stabilized by crown ether‐coordinated K^+^ cations. The [K(18‐crown‐6)]^+^ salts have been isolated as solids and are thus significantly more stable than the known [NMe_4_]^+^ derivatives. As a general trend, trifluoromethylated phosphoranides tend to decompose under formal liberation of difluorocarbene, giving rise to mixed fluoro(trifluoromethyl)phosphoranides. The typical reactivity of [P(CF_3_)_4‐n_F_n_]^−^ (n=0‐2) is determined by the tendency to donate one of the axial substituents, thus they serve as trifluoromethylating (n=0) and fluorinating agents (n=1‐2) which has been demonstrated by reactions with several different electrophiles. Moreover, the trivalent phosphoranides are oxidized by chlorine or Deoxo‐Fluor® to give pentavalent trifluoromethylphosphates. Solid‐state structures of [K(18‐crown‐6)][P(CF_3_)_4_] and [K(18‐crown‐6)][P(CF_3_)_3_F] have been elucidated. The examined phosphoranide anions exhibit a distorted trigonal bipyramidal geometry, where the sterically active lone pair leads to a decrease of the bond angle between the axial substituents. Elongated distances to the axial substituents are evident, due to the 3‐center‐4‐electron bonding. Finally, the solid‐state packing of the trifluoromethyl phosphoranides [K(18‐crown‐6)][P(CF_3_)_4_] and [K(18‐crown‐6)][P(CF_3_)_3_F] reveals zig‐zag chains, which are formed by an alternating arrangement of anions and cations, bridged by K−F interactions. Apart from that, variable temperature NMR experiments in different solvents disclose exchange processes in the phosphoranides.

## Conflict of interest

The authors declare no conflict of interest.

1

## Supporting information

As a service to our authors and readers, this journal provides supporting information supplied by the authors. Such materials are peer reviewed and may be re‐organized for online delivery, but are not copy‐edited or typeset. Technical support issues arising from supporting information (other than missing files) should be addressed to the authors.

Supporting Information

## Data Availability

The data that support the findings of this study are available from the corresponding author upon reasonable request.
