# Supplementary material for: Synthesis, Reactivity and Structural Properties of Trifluoromethylphosphoranides
Source: Chemistry. 2022 Feb 9;28(14):e202104308. doi: 10.1002/chem.202104308 (PMC9305102; doi:10.1002/chem.202104308)
Supplement: Supplementary file 1 — Supporting Information [file CHEM-28-0-s001.pdf]

Chemistry–A European Journal

Supporting Information

Synthesis, Reactivity and Structural Properties of Trifluoromethylphosphoranides

Oleg O. Shyshkov, Alexander A. Kolomeitsev, Berthold Hoge, Enno Lork, Axel Haupt,* Mira Keßler,* and Gerd-Volker Röschenthaler*

Table of Contents

1. General Information 1
2. Synthetic Procedures 2
Tris(trifluoromethyl)phosphine (**1**) 2
Tetrakis(trifluoromethyl)phosphoranide [K(18-crown-6)][$P(CF_3)_4$] (**2**) 2
Tetrakis(trifluoromethyl)phosphoranide [NMe_4][$P(CF_3)_4$] 3
Fluorotris(trifluoromethyl)phosphoranide [K(18-crown-6)][$P(CF_3)_3F$] (**3**) 3
Fluorotris(trifluoromethyl)phosphoranide [NMe_4][$P(CF_3)_3F$] 4
Difluorobis(trifluoromethyl)phosphoranide [NMe_4][$P(CF_3)_2F_2$] (**4**) 4
Attempted preparation of Trifluoro(trifluoromethyl)phosphoranide [NMe_4][$P(CF_3)F_3$] 4
3. Reactivity and Follow-up Chemistry 5
Pyrolysis of trifluoromethylphosphoranides 5
Hydrolysis of trifluoromethylphosphoranides 5
Reaction of trifluoromethylphosphoranides with Me_3SiCl 5
Reaction of [NMe_4][$P(CF_3)_2F_2$] (**4**) with Me_3SiCl 5
Reaction of [K(18-crown-6)][$P(CF_3)_4$] (**2**) with Me_3SiCF_3 6
Reaction of trifluoromethylphosphoranides with SO_2 6
Reaction of [K(18-crown-6)][$P(CF_3)_4$] (**2**) with 4-chlorobenzenesulfonyl chloride 6
Reaction of [K(18-crown-6)][$P(CF_3)_4$] (**2**) with benzaldehyde 7
Reaction of [K(18-crown-6)][$P(CF_3)_4$] (**2**) with trimethylborate 7
Reaction of [K(18-crown-6)][$P(CF_3)_4$] (**2**) with 2-fluoro-1,3-dimethylimidazolidinium triflate 7
Reaction of trifluoromethylphosphoranides with hexafluoroacetone 7
Oxidation of trifluoromethylphosphoranides by chlorine 8
Oxidation of trifluoromethylphosphoranides by $(MeOCH_2)_2NSF_3$ (Deoxo-Fluor®) 8
Reaction of trifluoromethylphosphoranides with methyl iodide 9
Reaction of [NMe_4][$P(CF_3)_4$] with methyl triflate 9
4. Additional data and visualizations for NMR-experiments 10
5. References 17

1. General Information

All reactions and manipulations were conducted under an atmosphere of dry nitrogen. The glassware was usually kept overnight in a drying oven at 160 °C and then cooled under a flow of dry nitrogen. Reactions with gases were carried out using standard vacuum techniques in a system made of Pyrex® glass and stopcocks lubricated with “Waker” medium duty silicon grease. Reactions were conducted in thick-walled round-bottom Pyrex® tubes with Teflon® stop-cocks. Nonvolatile air- and moisture-

sensitive materials were handled under inert atmosphere in a glove box “M. Braun Unilab 1200/780” with integrated fridge and gas purifying unit. The compounds were handled at room temperature, but stored in a fridge at −30 °C. The trifluoromethylated phosphorus compounds and other reagents required for our studies were prepared according to literature methods: CF_3PF_2[1], CF_3SiMe_3[2], NMe_4F[3]. Solid fluorides and solvents were purified, dried and degassed by standard methods. The following commercially available chemicals were used without further purification: $(PhO)_3P$, 18-crown-6, KF, CH_3I, $(CF_3)_2CO$, $ClC_6H_4SO_3Cl$, $CH_3C_6H_4SO_3Cl$, Cl_2, HCl, $(CH_3OCH_2)_2NSF_3$ (“Deoxo-Fluor®”, generous gift of *Hansa Fine Chemicals GmbH*), SO_2, PhCHO.

Mass spectrometric analysis (70 eV) was carried out on a MAT 8200 type spectrometer (Varian MAT) as well as by FAB measurements. High resolution mass spectra were recorded on a Finnigan MAT 8222 spectrometer using Peak-Matching Method. The X-ray structural study was carried out on a Siemens P4 diffractometer using graphite monochromated Mo-K_α radiation (λ = 71.073 pm). While measuring, crystals were cooled down to −100 °C with the low-temperature nozzle Siemens LTII. The diffractometer was operated by XSCAnS program. NMR spectra were obtained on a Bruker DPX-200 spectrometer operating at 200 MHz for ^{1}H, 50 MHz for ^{13}C, 188 MHz for ^{19}F and 81 MHz for ^{31}P and on a Bruker AMX-360 spectrometer operating at 360 MHz for ^{1}H, 91 MHz for ^{13}C, 188 MHz for ^{19}F and 146 MHz for ^{31}P. Me_4Si was used as internal standard for ^{1}H and ^{13}C measurements, for ^{19}F and ^{31}P measurements CCl_3F and 85 % H_3PO_4 were used, respectively. Melting points were estimated on a Jürgens Electrothermal Melting Point Instrument with a working range from 20 °C to 360 °C. The temperatures are uncorrected. Elemental analyses were performed by Beller Microalanalytisches Laboratorium, Göttingen, Germany.

2. Synthetic Procedures

*Tris(trifluoromethyl)phosphine (**1**)*

CAUTION!!! $P(CF_3)_3$ is a highly flammable liquid with a low boiling point (17 °C). It readily explodes in liquid state upon contact with air. Sometimes, quite long induction times can be observed. Avoid leaks and use steel clamps for safety.

In a 300 ml thick-walled round-bottom Pyrex® tube with a Teflon® stop-cock triphenylphosphite (10 g; 32 mmol) and CF_3SiMe_3 (15.1 g; 106 mmol) were dissolved in anhydrous triglyme (50 ml) at 0 °C. Potassium phenolate (0.42 g; 3.2 mmol) was added to the cooled mixture. The reaction mixture was warmed to room temperature and then stirred at 50 °C for 2.5 h. After cooling, all volatiles were pumped off and collected in a flask. Using a Vigreux column, the volatiles were distilled at atmospheric pressure to give $P(CF_3)_3$ (6.1 g; 26 mmol; 81 %). NMR-spectra of the product were in accordance with literature data.[4]

*Tetrakis(trifluoromethyl)phosphoranide [K(18-crown-6)][P(CF$_3$)$_4$] (**2**)*

The reaction was carried out in a 250 ml Schlenk flask equipped with a magnetic stirring bar and connected to a vacuum line. KF (0.4 g; 6.9 mmol) was suspended in a solution of 18-crown-6 (2.0 g; 7.6 mmol) in 40 ml of dry monoglyme. Subsequently, Me_3SiCF_3 (1.1 g; 7.6 mmol) and $(CF_3)_3P$ (2.6 g; 11 mmol) were condensed to the mixture. The reaction was quickly warmed to 0 °C and allowed to reach room temperature under intensive stirring for 45 minutes. Then, 2/3 of the solvent were evaporated as quickly as possible at a reduced pressure of 0.01 mmHg while warming the reaction

vessel by a cold water-bath (10 °C to rt). The suspension obtained was cooled to −30 °C and the product was precipitated and washed with anhydrous ether (100 ml for precipitation + 2×10 ml for washing, filtration at rt). The residual ether was removed in high vacuum (5 min at rt) to give a white fluffy powder (3.87 g; 6.33 mmol; 92 % yield; 93 % purity) which was stored at −40 °C under inert atmosphere.
Alternatively, analytically pure **2** is obtained in lower yield (62 %), if monoglyme is not evaporated from the reaction mixture. In this case, the phosphoranide is precipitated by addition of at least 200 ml of dry ether at −30 °C. The solid is filtered under nitrogen and washed with ether at rt to remove excess 18-crown-6. The rather high solubility of **2** in ether/monoglyme leads to a partial loss of substance.

$C_{16}H_{24}F_{12}KO_6P$, FW = 610.41 g/mol, m.p. = 140 °C(dec); ^1H NMR (THF-d8, 293 K): δ/ppm = 3.8 (s); ^{19}F NMR (THF-d8, 223 K): δ/ppm = −65.4 (sep.d, CF_{ax}, 6F, $^2J_{F,P}$ = 2 Hz, $^4J_{F,F}$ = 8 Hz), −53.9 (d.sep, CF_{eq}, 6F, $^2J_{F,P}$ = 69 Hz, $^4J_{F,F}$ = 8 Hz); ^{31}P NMR (THF-d8, 223 K): δ/ppm = −50 (br.sep, $^2J_{F,P}$ = 69 Hz); Anal. Calcd. for brutto formula: C, 31.48; H, 3.96; F, 37.35; P, 5.07; Found: C, 31.64; H, 3.89; F, 38.15; P, 5.21.

Tetrakis(trifluoromethyl)phosphoranide [NMe₄][P(CF₃)₄]

The reaction was carried out in a Pyrex® λ-tube equipped with a Teflon® stop-cock and a magnetic stirring bar. Me_3SiCF_3 (0.8 g; 5.6 mmol) and $P(CF_3)_3$ (1.9 g; 8.1 mmol) were condensed to a suspension of NMe_4F (0.5 g; 5.4 mmol) in anhydrous diethyl ether (15 ml). The mixture was quickly warmed to −55 °C and was left stirring at this temperature for 8 h. The precipitate was washed three times applying standard λ-tube techniques (decantation of the mother liquor and recondensation of the solvent to the precipitate at −55 °C). Finally, the solvent was evaporated in high vacuum at −30 °C during 30 min to give $[NMe_4][P(CF_3)_4]$ as a slightly yellow powder in almost quantitative yield (2.0 g; 5.3 mmol; 98 %). The phosphoranide obtained was noticed to explode spontaneously and therefore should be handled carefully.

$C_8H_{12}F_{12}NP$, FW = 381.14 g/mol, m.p. = 93 °C(dec); ^1H NMR (THF-d8, 223 K): δ/ppm = 3.1 (s); $^{13}C\{^{19}F\}$ NMR (Monoglyme, int. Toluene-d8, 223 K): δ/ppm = 129.5 (d, CF_{ax}, $^1J_{C,P}$ = 73 Hz), 135.8 (d, CF_{eq}, $^1J_{C,P}$ = 195 Hz); ^{19}F NMR (Monoglyme, int. Toluene-d8, 223 K): δ/ppm = −63.0 (sep. d, CF_{ax}, 6F, $^2J_{F,P}$ = 3 Hz, $^4J_{F,F}$ = 9 Hz), −51.7 (d.sep, CF_{eq}, 6F, $^2J_{F,P}$ = 69 Hz, $^4J_{F,F}$ = 9 Hz); ^{31}P NMR (Monoglyme, int. Toluene-d8, 223K): δ/ppm = −49.5 (br.sep, $^2J_{F,P}$ = 69 Hz); Anal. Calcd. for brutto formula: C, 25.21; H, 3.17; F, 59.82; N, 3.67; P, 8.13; Found: C, 25.28; H, 3.10; F, 59.36; N, 3.62; P, 8.24.

*Fluorotris(trifluoromethyl)phosphoranide [K(18-crown-6)][P(CF₃)₃F] (**3**)*

The reaction was carried out in a 250 ml Schlenk flask equipped with a magnetic stirring bar and connected to a vacuum line. KF (0.5 g; 8.6 mmol) was suspended in a solution of 18-crown-6 (2.9 g; 11 mmol) in dry diethyl ether (70 ml). Subsequently, $(CF_3)_3P$ (4.1 g; 17 mmol) was condensed to the mixture. The reaction was quickly warmed to room temperature and stirred for 16 hours. Heavy but mobile grains of KF were gradually substituted by friable flakes of the phosphoranide formed. The solid was filtrated under nitrogen, washed with dry ether (20 ml) and dried in high vacuum (10 min at rt) to give a white fluffy powder (4.73 g; 8.43 mol; 98 % yield; 99 % purity).

$C_{15}H_{24}F_{10}KO_6P$, FW = 560.40 g/mol, m.p. = 147 °C; ^1H NMR (THF-d8, 293 K): δ/ppm = 3.8 (s); ^{19}F NMR (THF-d8, 183 K): δ/ppm = −59.3 (br.s, $CF_{3(eq+ax)}$, 9F, Δν½ = 304 Hz), −19.8 (br.s, F_{ax}, 1F, Δν½= 33 Hz); ^{31}P NMR (THF-d8, 183 K): δ/ppm = −61.9 (br.dec); Anal. Calcd. for brutto formula: C, 32.15; H, 4.32; F, 33.90; P, 5.53; Found: C, 32.22; H, 4.01; F, 33.97; P, 5.50.

Fluorotris(trifluoromethyl)phosphoranide $[NMe_4][P(CF_3)_3F]$

The reaction was carried out in a Pyrex® λ-tube equipped with a Teflon® stop-cock and a magnetic stirring bar. $(CF_3)_3P$ (1.9 g; 8.1 mmol) was condensed to a suspension of NMe_4F (0.5 g; 5.4 mmol) in dry ether (15 ml). The mixture was quickly warmed to −40 °C and was left stirring in a temperature range between −50 and −40 °C for 8 h. The precipitate was washed three times applying standard λ-tube techniques (decantation of the mother liquor and recondensation of the solvent to the precipitate at −40 °C). Finally, the solvent was evaporated in high vacuum at −20 to −10 °C during 30 min to give $[NMe_4][P(CF_3)_3F]$ as a slightly yellow powder (1.71 g; 5.16 mmol; 95 %).

$C_7H_{12}F_{10}NP$, FW = 331.14 g/mol, m.p. = 127 – 129 °C(dec); ^{1}H NMR (THF-d8, 223 K): δ/ppm = 3.1 (s); ^{19}F NMR (Monoglyme, int. Toluene-d8, 223 K): δ/ppm = −61.2 (d.d.sept, CF_{ax}, 3F, $^2J_{F,P}$ = 29 Hz, $^3J_{F,F}$ = 38 Hz, $^4J_{F,F}$ = 10 Hz), −54.0 (d.d.q, CF_{eq}, 6F, $^2J_{F,P}$ = 85 Hz, $^3J_{F,F}$= 11 Hz, $^4J_{F,F}$ = 10 Hz), −14.1 (d.q.sept, F_{ax}, 1F, $^1J_{F,P}$ = 385 Hz, $^3J_{F,F}$ = 38 / 11 Hz); ^{31}P NMR (Monoglyme, int. Toluene-d8, 223 K): δ/ppm = −54.9 (d.sep.q, $^1J_{F,P}$ = 385 Hz, $^2J_{F(eq),P}$ = 85 Hz, $^2J_{F(ax),P}$ = 29 Hz); ^{13}C/^{19}F COSY NMR (Monoglyme, int. Toluene-d8, 223 K): δ/ppm = 56.9 ((H_3**C**)N), 130.4 (CF_{3eq}), 136.6 (CF_{3ax}); Anal. Calcd. for brutto formula: C, 25.39; H, 3.65; F, 57.37; N, 4.23; P, 9.35; Found: C, 25.71; H, 3.27; F, 57.65; N, 4.00; P, 9.01.

*Difluorobis(trifluoromethyl)phosphoranide $[NMe_4][P(CF_3)_2F_2]$ (**4**)*

NMe_4F (0.5 g; 5.4 mmol) was suspended in dry ether (20 ml) placed in a 300 ml thick-walled round-bottom Pyrex® tube with a Teflon® stop-cock. CF_3PF_2 (1.85 g; 13.4 mmol) was condensed to this mixture. Dry nitrogen was added to the tube until atmospheric pressure was present, then the tube was closed and quickly warmed to room temperature and stirred for 16 h. The white precipitate was filtered under nitrogen, washed with dry ether (10 ml) and dried in high vacuum (10 min at rt) to give a white fluffy powder (1.47 g; 5.24 mol; 97.6 % yield; 99 % purity).

$C_6H_{12}F_8NP$, FW = 281.13 g/mol, m.p. = 172 °C(dec); ^{1}H NMR (THF-d8, 223 K): δ/ppm = 3.11 (s); ^{13}C NMR (Monoglyme, int. Toluene-d8): δ/ppm = 56.9 (s, (H_3**C**)N), 128.6 (m, CF); ^{19}F NMR (Monoglyme, int. Toluene-d8): δ/ppm = −63.9 (d.t, CF_{eq}, 6F, $^2J_{F,P}$ = 94 Hz, $^3J_{F,F}$ = 14 Hz), −69.2 (d.sep, F_{ax}, 2F, $^1J_{F,P}$ = 312 Hz, $^3J_{F,F}$ = 14 Hz); ^{31}P NMR (Monoglyme, int. Toluene-d8): δ/ppm = −10.4 (t.sep, $^1J_{F,P}$ = 312 Hz, $^2J_{F,P}$ = 94 Hz); Anal. Calcd. for brutto formula: C, 25.63; H, 4.30; F, 54.06; N, 4.98; P, 11.02; Found: C, 26.64; H, 4.42; F, 54.37; N, 5.01; P, 11.07.

Attempted preparation of Trifluoro(trifluoromethyl)phosphoranide $[NMe_4][P(CF_3)F_3]$

A Pyrex® NMR tube with a Teflon® stop-cock was charged with NMe_4F (0.03 g; 0.32 mmol) and attached to a vacuum line. Anhydrous acetonitrile (1.5 ml) and CF_3PF_2 (0.049 g, 0.352 mmol) were condensed to the tube, which was then carefully warmed to −38 °C and immediately submitted to low-temperature NMR experiments. When removed from the NMR spectrometer at room temperature, the sample turned dark-yellow and contained some solid matter on the walls of the tube – probably reaction products of hypervalent phosphorus species (or products of their decomposition) with the solvent.

$C_5H_{12}F_6NP$, FW = 231.12 g/mol; ^{31}P NMR (CH_3CN, 235 K): δ/ppm = −10.4 (t.d.q., $^1J_{F(ax),P}$ = 450 Hz, $^1J_{F(eq),P}$ = 1237 Hz, $^2J_{F,P}$ = 80 Hz).

3. Reactivity and Follow-up Chemistry

Pyrolysis of trifluoromethylphosphoranides

A heavy-walled round-bottom 150 ml Pyrex® tube with a Teflon® stop-cock was charged with 2 to 5 mmol of the corresponding phosphoranide and slowly warmed until decomposition of the compound, which typically occurred before melting. After finished decomposition, the temperature was slowly raised until complete degradation of the tube content, which was accompanied by liquefying, colour changes and gas evolution. The evolved gas was collected in a Pyrex® NMR tube equipped with a Teflon® stop-cock and filled with a suitable solvent. Both gaseous and solid pyrolysis products were analyzed by NMR spectroscopy. The results are summarized in the following table:

**Phosphoranide**	**Decomposition [°C]**	**Melting Point [°C]**	**Pyrolysis [°C]**	**Main Products of Pyrolysis**
$[NMe_4][P(CF_3)_4]$	60	93 (gas evolution)	rt to 53 (spontaneously, explosion)	PF_3, CF_3H, NMe_3
$[NMe_4][P(CF_3)_3F]$	108	127-129 (gas evolution)	104 (explosion)	PF_3, $P(CF_3)_3$, CF_3H, NMe_3
$[NMe_4][P(CF_3)_2F_2]$ (**4**)	82	172 (gas evolution at 184)	84 to 90 (spontaneously, explosion)	PF_3, CF_3H, NMe_3
[K(18-crown-6)] $[P(CF_3)_4]$ (**2**)	110	140-144 (gas evolution)	138-142	$P(CF_3)_3$
[K(18-crown-6)] $[P(CF_3)_3F]$ (**3**)	138 (becomes slightly yellow)	147 (gas evolution and rapid darkening at 157)	160	$P(CF_3)_3$

Hydrolysis of trifluoromethylphosphoranides

The phosphoranide salt (2 to 5 mmol) was dissolved in dry monoglyme (15 ml) and cooled to −20 °C. Degassed water (35 mmol) was added to the solution and the mixture was slowly warmed to room temperature. Hydrolysis products were analyzed by NMR spectroscopy and mass spectrometry.

$[NMe_4][PH(CF_3)(=O)O]$: $C_5H_{13}F_3NO_2P$, FW = 207.13 g/mol; ^{19}F NMR (Monoglyme/H_2O): δ/ppm = −79.1 (d.d, CF_3, $^{2}J_{F,P}$ = 96 Hz, $^{3}J_{H,F}$ = 4 Hz); ^{31}P NMR (Monoglyme/H_2O): δ/ppm = 2.0 (d.q, $^{1}J_{H,P}$ = 561 Hz). Data are in good agreement with the literature.[5]

Reaction of trifluoromethylphosphoranides with Me_3SiCl

A Pyrex® NMR tube with a Teflon® stop-cock was charged with 0.2 to 0.4 mmol of the corresponding phosphoranide salt and attached to a vacuum line. Dry monoglyme (1.5 ml) and a 4-fold excess of Me_3SiCl were condensed to the phosphoranide. The tube was warmed to room temperature and submitted to NMR experiments.
Depending on the initial phosphoranide, signals of Me_3SiF or Me_3SiCF_3 were revealed in the NMR spectra of the reaction mixture and their relative intensities were in a proper ratio with that of $P(CF_3)_3$ (molar ratio 1/1).

*Reaction of $[NMe_4][P(CF_3)_2F_2]$ (**4**) with Me_3SiCl*

A 50 ml Schlenk flask was charged with **4** (0.5 g; 1.8 mmol) and cooled to −30 °C. Dry monoglyme (5 ml) and Me_3SiCl (0.19 g; 1.8 mmol) were added to the substrate. The reaction mixture was kept at −30 °C for 3 h under constant stirring. A sample of 1 ml was taken for NMR investigation at −60 °C and the rest was concentrated in high vacuum. The product was precipitated by addition of cold ether, filtered and dried to give $[NMe_4][P(CF_3)_2ClF]$ (0.33 g; 1.11 mmol; 78 %).

$C_6H_{12}ClF_7NP$, FW = 297.58 g/mol; ^{19}F NMR (Monoglyme, 213 K): δ/ppm = −87.9 (d.sep, $F_{(ax)}$, 1F, $^{1}J_{PF}$= 504 Hz, $^{3}J_{F,F}$ = 10 Hz), −60.4 (d.d, $CF_{3(eq)}$, 6F, $^{2}J_{F,P}$ = 60 Hz, $^{3}J_{F,F}$ = 10 Hz); ^{31}P NMR (Monoglyme, 213 K): δ/ppm = 12.0 (d.q, $^{1}J_{PF}$= 504 Hz, $^{2}J_{F,P}$ = 60 Hz). Data are in good agreement with the literature.[6]

*Reaction of [K(18-crown-6)][P(CF$_3$)$_4$] (**2**) with Me$_3$SiCF$_3$*

A Pyrex® NMR tube with a Teflon® stop-cock was charged with **2** (0.12 g; 0.20 mmol) and attached to a vacuum line. Dry monoglyme (1.5 ml) and Me_3SiCF_3 (0.043 g, 0.3 mmol) were condensed into the tube. The mixture was carefully warmed to −55 °C and immediately submitted to low-temperature NMR measurements (−55 °C to rt).

^{19}F NMR spectra of the reaction mixture: (Monoglyme, 233 K): δ/ppm = −64.3 (sep.d, $CF_{3(ax)}$, $^{2}J_{F,P}$ = 2 Hz), −52.9 (d.sep, $CF_{3(eq)}$, $^{2}J_{F,P}$ = 68 Hz, $^{4}J_{F,F}$ = 8 Hz).

Reaction of trifluoromethylphosphoranides with SO$_2$

A Pyrex® NMR tube with a Teflon® stop-cock was charged with 0.3 mmol of the corresponding phosphoranide and attached to a vacuum line. Then, 20 mmol of SO_2 were condensed into the tube. The mixture was quickly warmed to −78 °C and slowly from −78 °C to room temperature within 1.5 h.

*Reaction of **2** with SO$_2$ - spectra of the reaction mixture*: ^{19}F NMR (SO_2, 293 K): δ/ppm = −77.2 (br.s, [NMe$_4$][SO$_2$(C**F**$_3$)]), −50.7 (d, P(C**F**$_3$)$_3$, $^{2}J_{F,P}$ = 82 Hz); ^{31}P NMR (SO_2, 293 K): δ/ppm = −0.7 (dec, P(C**F**$_3$)$_3$, $^{2}J_{F,P}$ = 82 Hz). After removing all volatiles and dissolution of the residue in DMF a signal at δ/ppm = −87 ppm was revealed in the ^{19}F NMR spectrum, which is in good agreement with the literature for $[SO_2(CF_3)]^-$.[7]

*Reaction of **3** (or its analogous [NMe$_4$]$^+$ salt) with SO$_2$ - spectra of the reaction mixture*: only signals corresponding to $P(CF_3)_3$ were found in NMR spectra.

Reaction of [NMe$_4$][P(CF$_3$)$_3$F] with SO$_2$ – detection of [NMe$_4$][SO$_2$F]

A thick-walled round-bottom 150 ml Pyrex® tube with a Teflon® stop-cock was charged with $[NMe_4][P(CF_3)_3F]$ (1 g; 3 mmol) and SO_2 (5 g; 0.1 mol) was condensed into the tube. The reaction mixture was warmed to −30 °C within 40 min and stirred for 30 min at this temperature. Excess SO_2 was carefully evaporated in vacuum (approx. 20 mmHg, −30 °C). The residual creamy solid (0.45 g; 2.9 mmol; 98 %) was dissolved in CD_3CN at −30 °C and submitted to low-temperature NMR measurements.

Reaction of [NMe$_4$][P(CF$_3$)$_3$F] with SO$_2$ – spectrum of the solid obtained: ^{1}H NMR (CD_3CN, 243 K): δ/ppm = 3.1 (s); ^{19}F NMR (CD_3CN, 243K): δ/ppm = 103.0 (br.s). NMR data are in good agreement with the literature.[8]

*Reaction of [K(18-crown-6)][P(CF$_3$)$_4$] (**2**) with 4-chlorobenzenesulfonyl chloride*

A standard NMR tube was charged with **2** (0.18 g; 0.30 mmol) and cooled to −100 °C. Monoglyme (1.2 ml) and sulfonyl chloride (0.13 g; 0.60 mmol) were added to the tube under inert atmosphere. The tube was closed and the mixture was warmed to −78 °C and then from −78 °C to room temperature within 1.5 h (or within 5 min), while shaking the tube periodically.

*Reaction of **2** with 4-chlorobenzenesulfonyl chloride (quick warming to rt) - spectra of the reaction mixture*: ^{19}F NMR (Monoglyme, 293 K): δ/ppm = −51.9 (br.d, P(C**F**$_3$)$_3$, $^{2}J_{F,P}$ = 80 Hz), −29.7 (br.s, CF_3Cl); ^{31}P NMR (Monoglyme, 293 K): δ/ppm = −3.0 (br.dec, P(C**F**$_3$)$_3$, $^{2}J_{F,P}$ = 80 Hz); NMR yield: 100 %. Literature for CF_3Cl: ^{19}F NMR ($CDCl_3$): δ/ppm = −33.[9]

Reaction of **2** *with 4-chlorobenzenesulfonyl chloride (slow warming to rt) - spectra of the reaction mixture*: ^{19}F NMR (Monoglyme, 293 K): δ/ppm = −76.4 (s, *4*-Cl-$C_6H_4SO_2CF_3$), −51.3 (br.d, P(C**F**$_3$)$_3$, $^{2}J_{F,P}$ = 81 Hz) and signals of other unidentified compounds; ^{31}P NMR (Monoglyme, 293 K): δ/ppm = −2.9 (br.dec, P(C**F**$_3$)$_3$, $^{2}J_{F,P}$ = 81 Hz); NMR yield: 32 %. Literature for *4*-Cl-$C_6H_4SO_2CF_3$: ^{19}F NMR ($CDCl_3$): δ/ppm = −75.0.[10]

*Reaction of [K(18-crown-6)][P(CF$_3$)$_4$] (**2**) with benzaldehyde*

Benzaldehyde (0.11 g; 1.0 mmol) was added to a solution of **2** (0.5 g; 0.8 mmol) in dry monoglyme (7 ml) at −40 °C and the reaction mixture was stirred at this temperature for 30 min. Several drops of concentrated aqueous HCl were added to the mixture and its temperature was allowed to rise to 25 °C. The reaction product was not isolated but unambiguously identified by NMR spectroscopy.
PhCH(CF$_3$)(OH): $C_8H_7F_3O$, FW = 176.14 g/mol; ^{19}F NMR (Monoglyme, 293 K): δ/ppm = −79.4 (br.d, CF_3, $^{3}J_{H,F}$ = 7 Hz); NMR yield: 77 %. Literature: ^{19}F NMR ($CDCl_3$): δ/ppm = −79.0, $^{3}J_{H,F}$ = 7 Hz.[11]

*Reaction of [K(18-crown-6)][P(CF$_3$)$_4$] (**2**) with trimethylborate*

To a stirred solution of **2** (0.5 g, 0.8 mmol) in dry monoglyme (10 ml), B(OMe)$_3$ (0.1 g, 1.0 mmol) was added at −70 °C. The reaction mixture was kept at that temperature for 1 h and then allowed to warm to 25 °C. The reaction product was not isolated but unambiguously identified by NMR spectroscopy and mass spectrometry.
[K(18-crown-6)][B(OMe)$_3$(CF$_3$)]: $C_{16}H_{33}BF_3KO_9$, FW = 476.33 g/mol; ^{11}B NMR (Monoglyme, 293 K): δ/ppm = −0.9 (q, $^{2}J_{B,F}$ = 30 Hz); ^{19}F NMR (Monoglyme, 293 K): δ/ppm = −71.1 (m, CF_3). NMR yield: 72 %. Literature: ^{11}B NMR (D_2O): δ/ppm = −0.9, q, $^{2}J_{B,F}$ = 30 Hz; ^{19}F NMR (D_2O): δ/ppm = −75.9, m.[12]

*Reaction of [K(18-crown-6)][P(CF$_3$)$_4$] (**2**) with 2-fluoro-1,3-dimethylimidazolidinium triflate*

To a stirred solution of **2** (0.5 g, 0.8 mmol) in dry monoglyme (10 ml), 2-fluoro-1,3-dimethylimidazolidinium triflate (0.27 g, 1.0 mmol) was added at −40 °C. The reaction mixture was kept at that temperature for 2 h and then allowed to warm to 25 °C within 30 min. The reaction product was not isolated but unambiguously identified by NMR spectroscopy and mass spectrometry.

2-fluoro-1,3-dimethyl-2-(trifluoromethyl)imidazolidine: $C_6H_{10}F_4N_2$, FW = 186.15 g/mol; ^{19}F NMR (Monoglyme, 293 K): δ/ppm = −72.1 (s, NCF, 1F); −63.6 (s, CF_3, 3F), MS: (EI, 70 eV, 200 °C) m/z (%): 186 (15) M$^+$, 167 (47) [M-F]$^+$, 136 (9) [M-CF_2]$^+$, 117 (100) [M-CF_3]$^+$, 69 (17) [CF_3]$^+$, and other fragments. NMR yield: 90 %.

Under the same conditions, phosphoranide **3** (or its analogous [NMe_4]$^+$ salt) reacts with the triflate salt mentioned above to produce DFI in nearly quantitative yield (evaluated by ^{19}F NMR) accompanied by traces of unidentified compounds.

Reaction of trifluoromethylphosphoranides with hexafluoroacetone

A thick-walled round-bottom 250 ml Pyrex® tube with a Teflon® stop-cock was charged with the corresponding phosphoranide (1 mmol) and dry monoglyme (12 ml). Hexafluoroacetone (1.3 mmol) were condensed into the tube. The reaction mixture was warmed to −30 °C and stirred for 30 min, afterwards it was allowed to warm to 25 °C. The volatiles were pumped off *in vacuo* to give a slightly-brown solid residue.

Reaction of **2** *with hexafluoroacetone*: ^{1}H NMR (THF-d8, 293 K): δ/ppm = 3.8 (s); ^{19}F NMR (THF-d8, 293 K): δ/ppm = −76.4 (br.s, 9F, [(C**F**$_3$)$_3$CO]$^-$); Isolated yield: 96 %. Literature: ^{19}F NMR (acetone-d6): δ/ppm = −76.0, s.[13]

Reaction of $[NMe_4][P(CF_3)_3F]$ with hexafluoroacetone: ^{1}H NMR (THF-d8, 293 K): δ/ppm = 3.1 (s); ^{19}F NMR (THF-d8, 293 K): δ/ppm = −84.4 (br.s, 1F, $[(CF_3)_2\mathbf{F}CO]^-$), −83.1 (br.s, 6F, $[(C\mathbf{F}_3)_2FCO]^-$); Isolated yield: 99 % . Literature: ^{19}F NMR ($CDCl_3$): δ/ppm = −85.0; −83.5.[14]

Oxidation of trifluoromethylphosphoranides by chlorine

A thick-walled round-bottom 150 ml Pyrex® tube with a Teflon® stop-cock was used as a reaction vessel for these transformations. To a solution of the corresponding phosphoranide (1 mmol; pregenerated as described above, usually TMA salts) in dry monoglyme (15 ml), a strictly equimolar amount of Cl_2 was condensed. The reaction mixture was warmed to −50 °C and stirred at this temperature for 4 h. Then, the solvent was evaporated while warming the reaction vessel by cold water. The solid obtained was washed with dry ether (2x5 ml), filtered under nitrogen atmosphere and dried in high vacuum to give a moisture sensitive, white powder.

Reaction of $[NMe_4][P(CF_3)_4]$ with Cl_2, yielding $[NMe_4][P(CF_3)_4Cl_2]$: $C_8H_{12}Cl_2F_{12}NP$; FW = 452.05 g/mol., m.p. = >360 °C; ^{1}H NMR (THF-d8, 293 K): δ/ppm = 3.1 (s); ^{19}F NMR (THF-d8, 293 K): δ/ppm = −63.9 (br.d, CF_3, $^{2}J_{F,P}$ = 101 Hz); ^{31}P NMR (THF-d8, 293 K): δ/ppm = −167.8 (dec, $^{2}J_{F,P}$ = 101 Hz); MS: (FAB negative, NBA, 300 °C) m/z (%): 377 (44) $[M]^-$, 323 (100) [unidentified fragment]$^-$, 361 (14) $[M\text{-}Cl\text{+}F]^-$, 311 (12) $[M\text{-}Cl\text{-}CF_2\text{+}F]^-$, 273 (9) $[M\text{-}Cl\text{-}CF_2\text{-}2F\text{+}F]^-$, 223 (1) $[M\text{-}Cl\text{-}2CF_2\text{-}2F\text{+}F]^-$, 201 (18) [unidentified fragment]$^-$, 69 (7) $[CF_3]^-$ and other fragments; HRMS M^- calculated: 376.89325, found: 376.89334, R = 5000; Anal. Calcd. for brutto formula: C, 21.26; H, 2.68; Cl, 15.69; F, 50.43; N, 3.10; P, 6.85; Found: C, 21.31; H, 2.70; Cl, 15.54; F, 50.37; N, 3.12; P, 6.92; isolated yield: 99 %.

Reaction of $[NMe_4][P(CF_3)_3F]$ with Cl_2, yielding $[NMe_4][P(CF_3)_3Cl_2F]$: $C_7H_{12}Cl_2F_{10}NP$; FW = 402.04 g/mol.; m.p. = >360 °C, ^{19}F NMR (THF-d8, 293 K): δ/ppm = −85.9 (d.m, PF, $^{1}J_{F,P}$ = 836 Hz), −64.9 (d.sept, CF_3, $^{2}J_{F,P}$ = 154 Hz, $^{4}J_{F,F}$ = 13 Hz), −64.9 (d.q, CF_3, $^{2}J_{F,P}$ = 162 Hz, $^{4}J_{F,F}$ = 14 Hz), −62.1 to −66.6 (set of multiplets), −51.4 (d.m, PF, $^{1}J_{F,P}$ = 886 Hz), −36.0 (d.m, PF, $^{1}J_{F,P}$ = 943 Hz), −34.0 (d.m, PF, $^{1}J_{F,P}$ = 883 Hz), −2.3 (d.m, PF, $^{1}J_{F,P}$ = 982 Hz); ^{31}P NMR (THF-d8, 293 K): δ/ppm = 142.69 to 173.37 (m); MS: (ESI negative, CH_3CN, 350 °C) m/z (%): 327 (100) $[M]^-$, 311 (61) $[M\text{-}Cl\text{+}F]^-$, 273 (5) $[M\text{-}Cl\text{-}2F\text{+}F]^-$, 201 (7) $[M\text{-}Cl\text{-}CF_3\text{-}2F\text{+}F]^-$, and other fragments; Anal. Calcd. for brutto fomula: C, 20.91; H, 3.01; Cl, 17.64; F, 47.25; N, 3.48; P, 7.70; Found: C, 21.07; H, 3.07; Cl, 14.81; F, 49.62; N, 3.51; P, 7.85; isolated yield: 92 %. Mixture of isomers and probably $[NMe_4][P(CF_3)_3F_2Cl]$ as by-product.

Oxidation of trifluoromethylphosphoranides by $(MeOCH_2)_2NSF_3$ (Deoxo-Fluor®)

To a solution of **2** or **3** (1 mmol) in dry monoglyme (20 ml), a 6-fold excess of Deoxo-Fluo® was added at −35 °C. The reaction mixture was slowly warmed to room temperature and stirred for 1 h. A solid phase immediately formed after addition of the fluorinating agent. The precipitate gradually dissolved to produce a slightly-brown, clear solution at the end of the reaction. The solvent was removed *in vacuo* and the residual solid was washed with dry ether and dried at 0.01 mmHg to give a white powder.

$[K(18\text{-crown-}6)][P(CF_3)_3F_3]$: $C_{15}H_{24}F_{12}KO_6P$; FW = 598.40 g/mol.; meridional structure: ^{19}F NMR (Monoglyme, int. Toluene-d8): δ/ppm = −96.7 (d.m, 2F, PF_2 (F and F *trans*), $^{1}J_{F,P}$ = 862 Hz), −69.8 (d.m, 3F, CF_3, $^{2}J_{F,P}$ = 90 Hz), −68.5 (d.q.m, 6F, $P(CF_3)_2$ (CF_3 and CF_3 *trans*), $^{2}J_{F,P}$ = 126 Hz, $^{4}J_{F,F}$ = 12 Hz), −60.6 (d.m, 1F, PF, $^{1}J_{F,P}$ = 884 Hz); ^{31}P NMR (Monoglyme, int. Toluene-d8): δ/ppm = −157.5 (d.t.q.sept, $^{1}J_{F,P}$ = 862 / 884 Hz, $^{2}J_{F,P}$ = 126 / 90 Hz); facial structure: ^{19}F NMR (Monoglyme, int. Toluene-d8): δ/ppm = −78.9 (d.dec, 3F, PF_3, $^{1}J_{F,P}$ = 797 Hz, $^{4}J_{F,F}$ = 11 Hz), −65.7 (d.q, 9F, CF_3, $^{2}J_{F,P}$ = 92 Hz, $^{4}J_{F,F}$ = 11 Hz); ^{31}P NMR (Monoglyme, int. Toluene-d8): δ/ppm = −157.48 (q.dec, $^{1}J_{F,P}$ = 797 Hz, $^{2}J_{F,P}$ = 92 Hz); ratio of the isomers mer./fac. = 1/1.4; yield: 95 %.

[K(18-crown-6)][$P(CF_3)_4F_2$]: $C_{16}H_{24}F_{14}KO_6P$; FW = 648.41 g/mol., m.p. = >360 °C; *cis*-isomer: ^{1}H NMR (THF-d8, 293 K): δ/ppm = 3.6 (s.); ^{19}F NMR (THF-d8, 293 K): δ/ppm = −69.1 (d.m, 2F, PF_2, $^1J_{F,P}$ = 852 Hz), −63.8 (d.sept.m, 6F, CF_3, $^2J_{F,P}$ = 73 Hz, $^4J_{F,F}$ = 12 Hz), −62.6 (d.sept.m, 6F, CF_3, $^2J_{F,P}$ = 102 Hz, $^4J_{F,F}$ = 12 Hz); ^{31}P NMR (THF-d8, 293 K): δ/ppm = −165.4 (t.sept.sept, $^1J_{F,P}$ = 852 Hz, $^2J_{F,P}$ = 73 / 102 Hz); ^{Y}ield (in the mixture with [K(18-crown-6)][$P(CF_3)_3F_3$], determined by NMR spectroscopy): 21 %.

Reaction of trifluoromethylphosphoranides with methyl iodide

A Pyrex® NMR tube with a Teflon® stop-cock was charged with **2** (0.12 g; 0.20 mmol) and attached to a vacuum line. Dry monoglyme (1.5 ml) and MeI (0.23 g; 1.6 mmol) were condensed into the tube. The reaction mixture was quickly warmed to −30 °C and submitted to low temperature NMR measurements. Since it was revealed that the reaction occurs very slowly at 0 °C, the NMR tube was left at room temperature for 12 h and analyzed afterwards. The main reaction products are $P(CF_3)_3$ and CF_3CH_3 (^{19}F NMR (Monoglyme): δ/ppm = −62.0 (br.q, $^3J_{H,F}$ = 13 Hz)). Other unidentified products were also observed in ^{19}F and ^{31}P NMR spectra.

[NMe_4][$P(CF_3)_3F$] was pregenerated [NMe_4F (0.4 g; 4.3 mmol) + $(CF_3)_3P$ (1.3 g; 5.6 mmol) + monoglyme (30 ml) / 45 min at −15 °C] in a 250 ml two-neck round bottom flask connected to a funnel for filtration under inert atmosphere and equipped with a magnetic stirring bar and an adapter to the vacuum line. To the freshly prepared solution of the phosphoranide, MeI (6.1 g; 43 mmol) was condensed. The reaction mixture was quickly warmed to room temperature and stirred at this temperature for 30 min. An intensive formation of a creamy precipitate was revealed immediately after the temperature reached 10 to 15 °C. Then, 2/3 of the solvent were evaporated in vacuum and the residue was solidified (20 ml) and washed (7 ml) by the addition of dry ether. The obtained solid was dried in high vacuum to give a white powder.

[NMe_4][$P(CF_3)_3(CH_3)F_2$]: $C_8H_{15}F_{11}NP$; FW = 365.17 g/mol., m.p. = >360 °C; *cis-mer*-isomer (isolated as a powder): ^{1}H NMR (THF-d8, 293 K): δ/ppm = 1.2 (br.t, 3H, PCH_3, $^3J_{H,P}$ = 10 Hz), 3.2 (s, 12H, $N(CH_3)_4$); ^{13}C NMR (THF-d8, 293 K): δ/ppm = 124.95 to 135.84 (m); ^{19}F NMR (THF-d8, 293 K): δ/ppm = −66.8 (br.d.sept, 3F, CF_3, $^2J_{F,P}$ = 61 Hz, $^4J_{F,F}$ = 10 Hz), −64.5 (br.d.q.d.d, 6F, $P(CF_3)_2$ (CF_3 and CF_3 *trans*), $^2J_{F,P}$ = 88 Hz, $^4J_{F,F}$ = 10 Hz, $^3J_{F,F}$ = 15 / 15 Hz), −55.8 (d.m, 1F, PF, $^1J_{F,P}$ = 745 Hz), −50.5 (d.m, 1F, PF, $^1J_{F,P}$ = 773 Hz); ^{31}P{^{1}H} NMR (THF-d8, 293 K): δ/ppm = −165.0 (d.d.q.sept, $^1J_{F,P}$ = 773 / 745 Hz, $^2J_{F,P}$ = 88 / 61 Hz); MS: (FAB, negative, NBA, 300 °C) m/z (%): 290.8 (100) $[M]^-$, 220.9 (3) $[M\text{-}CF_3H]^-$ and other minor fragments; HRMS M^- calculated: 291.97972, found: 291.97987, R = 6000; isolated yield: 95 %; *trans-mer*-isomer or *cis-fac*-isomer (observed in solution): ^{19}F NMR (Monoglyme): δ/ppm = −65.3 (br.d.m, 6F, $^2J_{F,P}$ = 50 Hz), −62.6 (br.d.m, 3F, $^2J_{F,P}$ = 115 Hz), −45.3 (br.d., 2F, $^1J_{F,P}$ = 831 Hz); ^{31}P{^{1}H} NMR (Monoglyme): δ/ppm = −158.5 (br.m).

Reaction of [NMe_4][$P(CF_3)_4$] with methyl triflate

The glassware used to carry out this reaction was similar to that used above for the reaction with methyl iodide. $P(CF_3)_3$ (2.1 g; 8.7 mmol) and CF_3SiMe_3 (1.0 g; 7.0 mmol) were condensed to NMe_4F (0.6 g, 6.8 mmol) in dry monoglyme (15 ml). The mixture was warmed to −50 °C and stirred at this temperature for 2 h. Methyl triflate (1.9 g; 10.0 mmol) was added in one portion to the pale solution of [NMe_4][$P(CF_3)_4$] . The reaction mixture was warmed to −40 °C and stirred at this temperature for 2 h. Afterwards, the temperature was allowed to rise to 0 °C within 20 min and the reaction mixture was filtered under an atmosphere of nitrogen. The filtrate was evaporated, solidified and washed by dry ether (2×9 ml) to give *trans*-[NMe_4][$P(CF_3)_4(CH_3)F$] as a white powder (0.7 g; 1.7 mmol; 51 %).

trans-$[NMe_4][P(CF_3)_4(CH_3)F]$: $C_9H_{15}F_{13}NP$; FW = 415.16 g/mol., m.p. = 273 °C(dec.); ^1H NMR (THF-d8): δ/ppm = 1.3 (br.d, 3H, PCH_3), 3.1 (s, 12H, $N(CH_3)_4$); ^{13}C NMR (THF-d8): δ/ppm = 137.64 to 121.2 (m); ^{19}F NMR (THF-d8): δ/ppm = −49.6 (d.tridec.q, 1F, PF, $^1J_{F,P}$ = 753 Hz, $^3J_{F,F}$ = 11 Hz, $^3J_{H,F}$ ≈ 1 Hz), −65.5 (d.d., 12F, CF_3, $^2J_{F,P}$ = 73 Hz, $^3J_{F,F}$ = 11 Hz); $^{31}P\{^1H\}$ NMR (THF-d8): δ/ppm = −175.6 (d.tridec, $^1J_{F,P}$ = 753 Hz, $^2J_{F,P}$ = 73 Hz); MS: (FAB, negative, NBA, 300 °C) m/z (%): 340.9 (100) $[M]^-$; HRMS M^- calculated: 340.97910, found: 340.97907, R = 6000 ; isolated yield: 51.07 %

4. Additional data and visualizations for NMR-experiments

Figure S1 visualizes the behaviour of $[NMe_4][P(CF_3)_3F]$ in variable temperature ^{19}F NMR experiments. At −50 °C, intramolecular exchange processes are suppressed, so that three different types of fluorine atoms can be differentiated. In turn, heating to 80 °C leads to interconversion of the CF_3-groups, resulting in a broad signal in the trifluoromethyl region. Figure S2 shows the decomposition of $[NMe_4][P(CF_3)_3F]$ in monoglyme solution at room temperature. As can be seen by the decreasing intensity of the signal at about −55 ppm in the ^{31}P NMR spectra, which represents $[P(CF_3)_3F]^-$ (cf. Fig. S5), the phosphoranide steadily decomposes in solution at room temperature and is almost completely degraded after 12 days.

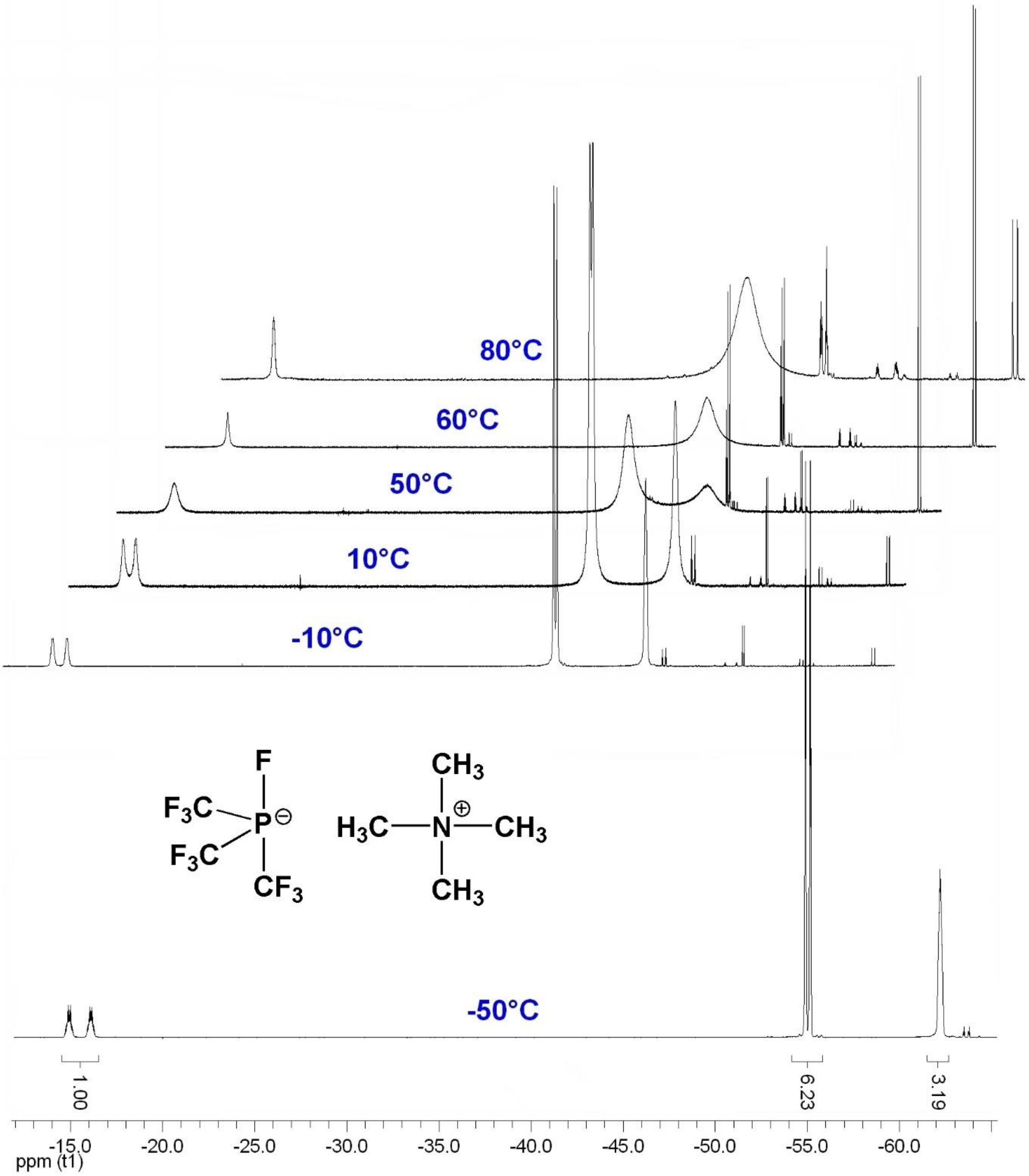

Fig. S1: Temperature dependent ^{19}F NMR spectra of $[NMe_4][P(CF_3)_3F]$ in monoglyme.

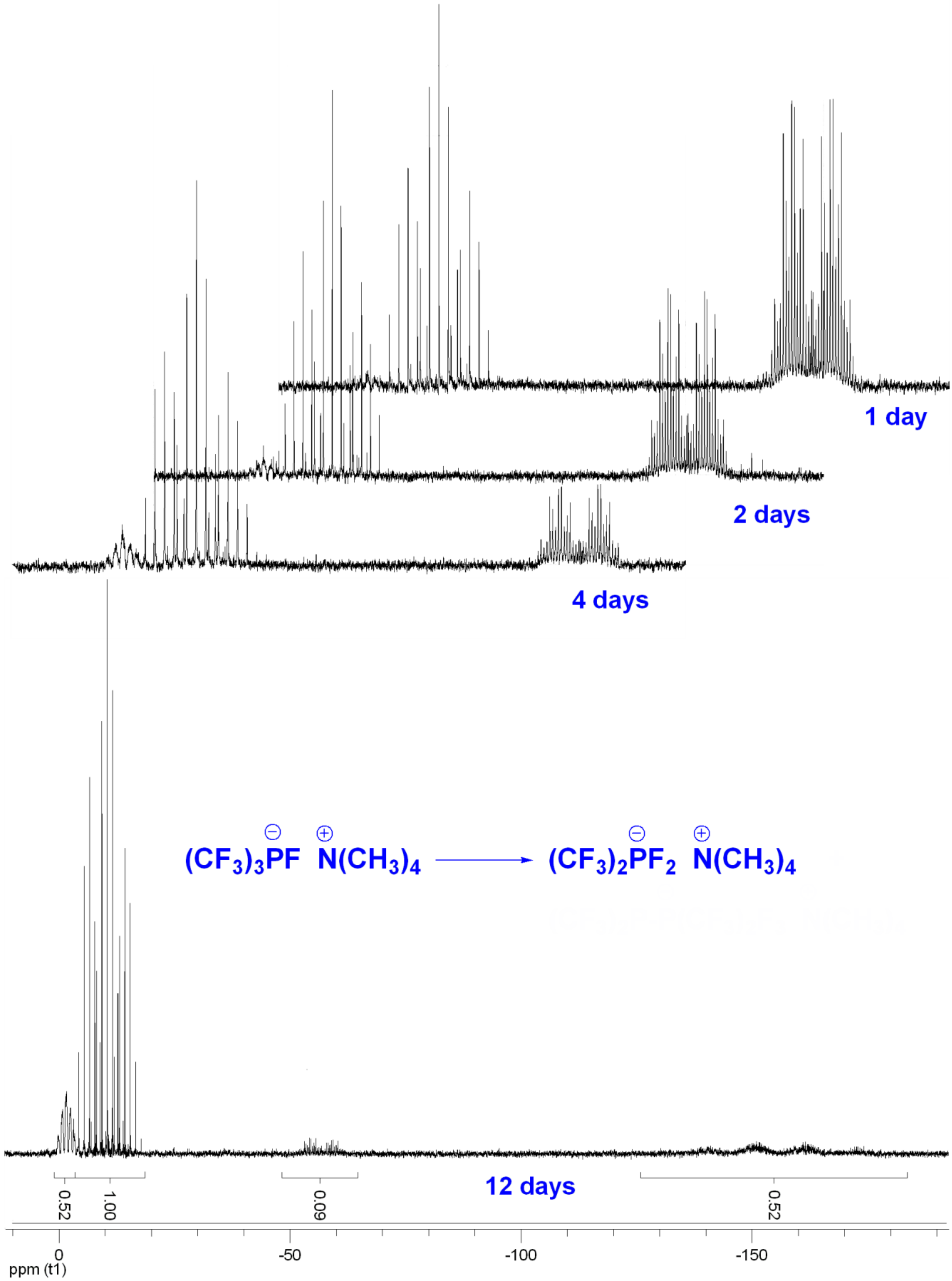

Fig. S2: Time dependent ^{31}P NMR spectra of $[NMe_4][P(CF_3)_3F]$ in monoglyme at room temperature.

Figures S3-S5 show the experimental ^{19}F and ^{31}P NMR resonances of $[NMe_4][P(CF_3)_3F]$ at −50 °C and the corresponding simulations, which have been obtained using gNMR.[15] With the help of the simulations it was possible to analyze the high-order NMR spectra for $[P(CF_3)_3F]^-$ and identify the following coupling constants: ^{1}J(P-F) = 385 Hz; ^{2}J(P-C$\mathbf{F}_{3eq.}$) = 85 Hz; ^{2}J(P-C$\mathbf{F}_{3ax.}$) = 29 Hz; ^{3}J(F-C$\mathbf{F}_{3eq.}$) = 11 Hz; ^{3}J(F-C$\mathbf{F}_{3ax.}$) = 38 Hz; ^{4}J(C$\mathbf{F}_{3ax.}$-C$\mathbf{F}_{3eq.}$) = 10 Hz. Figure S6 shows the ^{19}F NMR resonances of $[NMe_4][P(CF_3)_3F]$ in CD_3CN at room temperature.

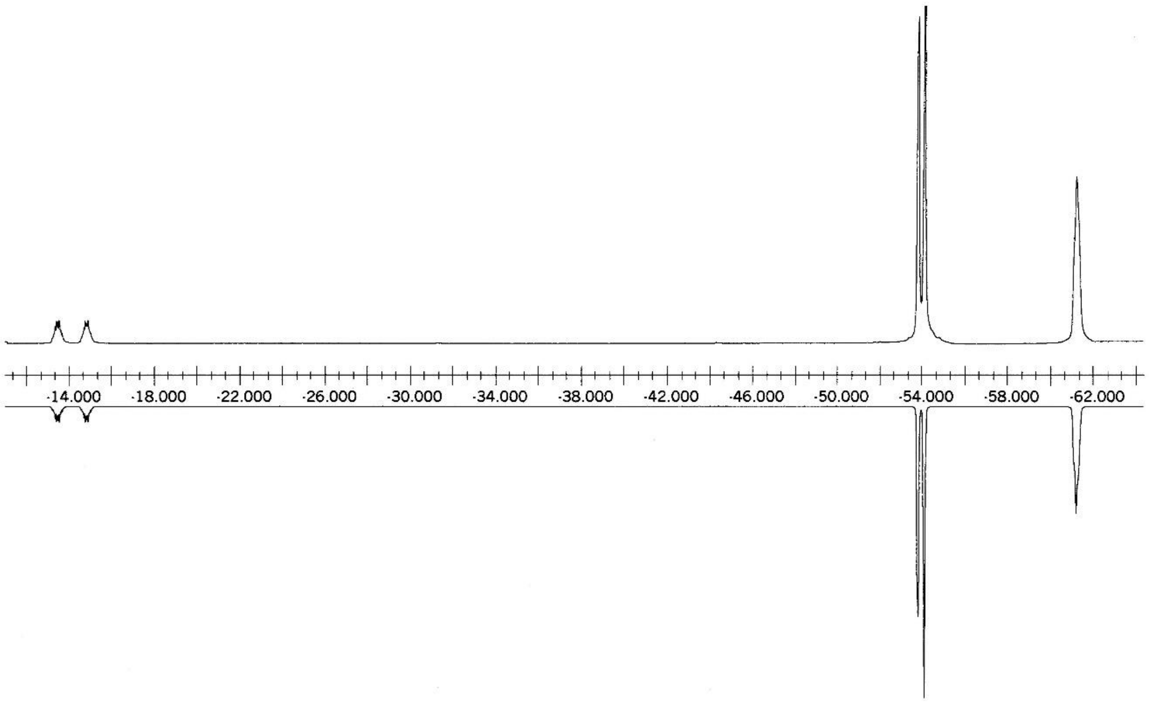

Fig. S3: Experimental (top) and simulated (bottom) ^{19}F NMR spectrum of $[NMe_4][P(CF_3)_3F]$ at −50 °C.

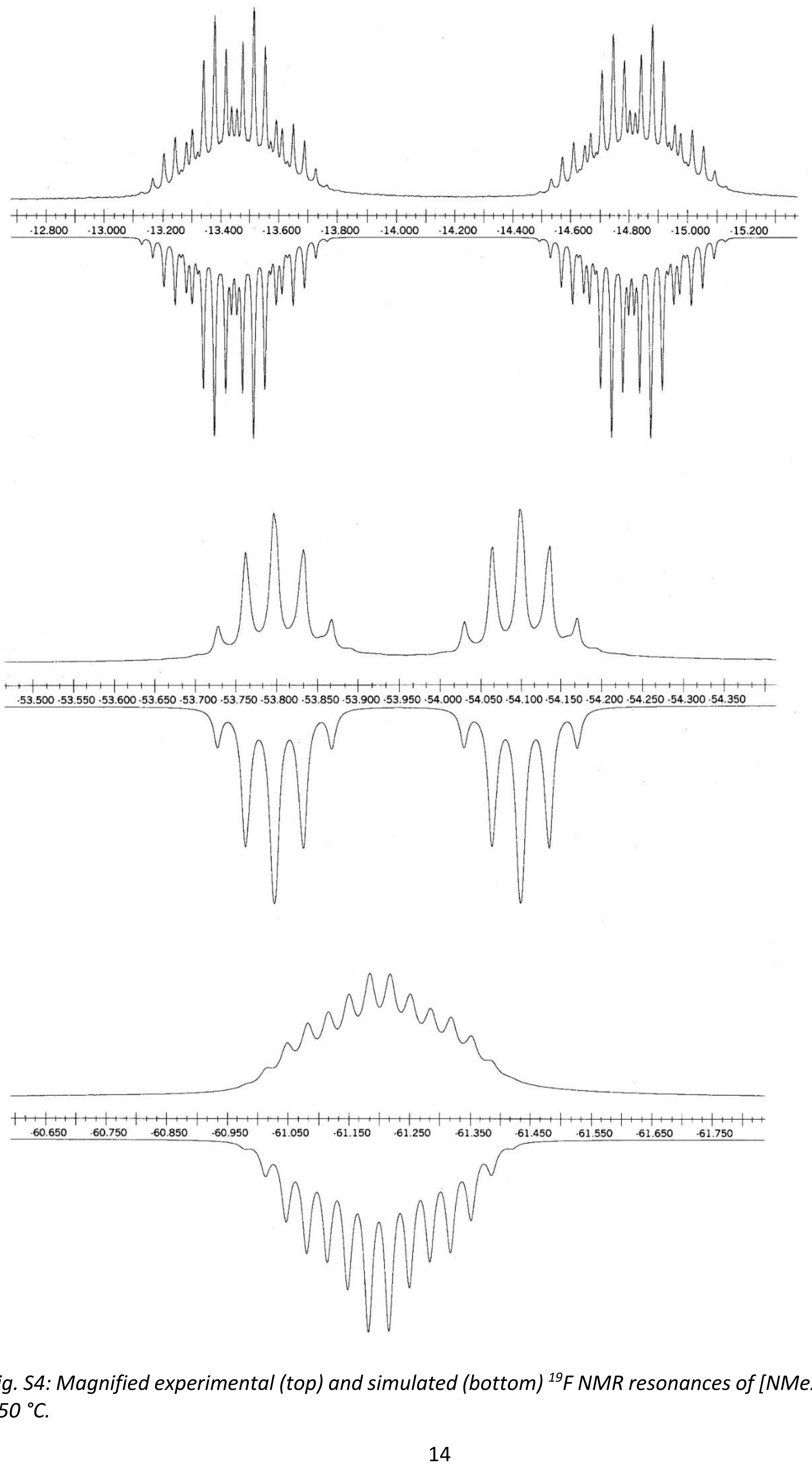

Fig. S4: Magnified experimental (top) and simulated (bottom) ^{19}F NMR resonances of $[NMe_4][P(CF_3)_3F]$ at −50 °C.

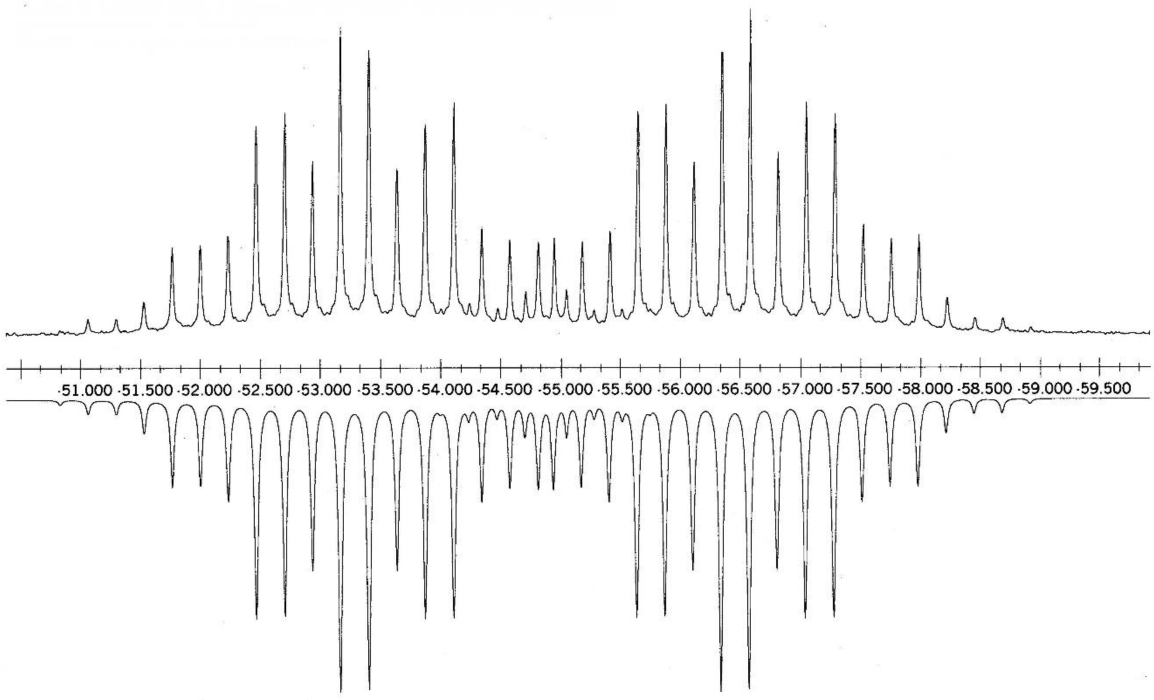

Fig. S5: Experimental (top) and simulated (bottom) ^{31}P NMR resonance of [NMe_4][$P(CF_3)_3F$] at −50 °C.

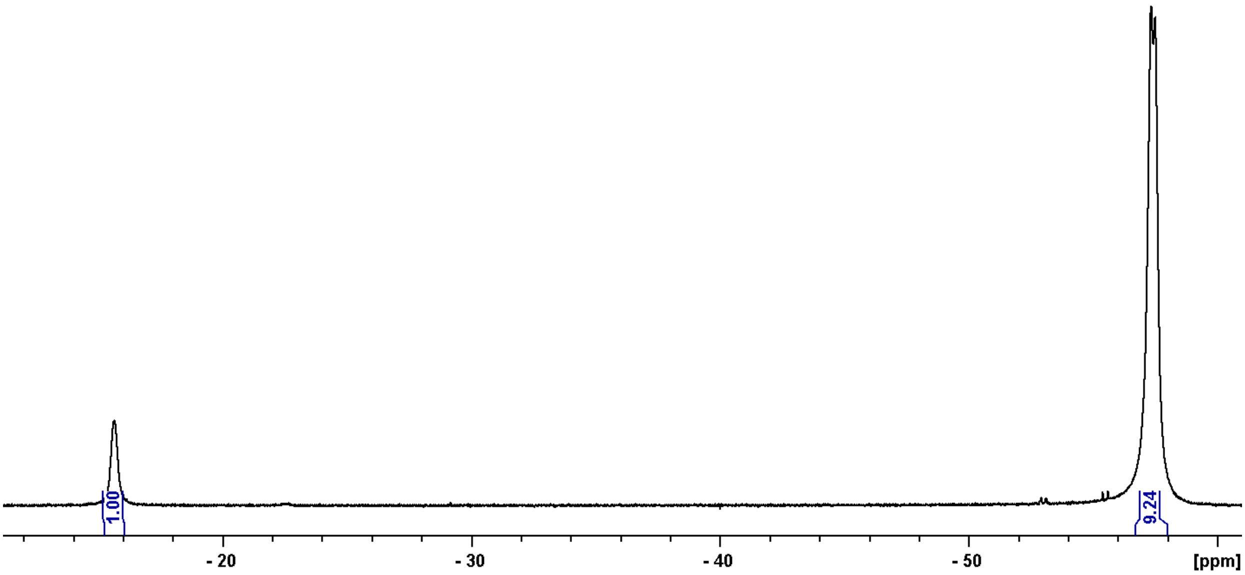

Fig. S6: ^{19}F NMR resonances of [NMe_4][$P(CF_3)_3F$] in CD_3CN at room temperature.

Figures S7 and S8 visualize temperature dependent NMR spectra of [K(18-crown-6)][P(CF_3)$_4$] (**2**) in monoglyme. As can be seen, two different types of CF_3-groups are observed at room temperature. Heating the sample leads to an interconversion of the trifluoromethyl groups, so that the two separate signals merge. At lower temperatures, fine splitting is observed for the CF_3-functions. Both the ^{19}F and ^{31}P NMR resonances of [K(18-crown-6)][P(CF_3)$_4$] (**2**) at −50 °C have been simulated and compared to the corresponding experimental spectra (Fig. S7).[15]

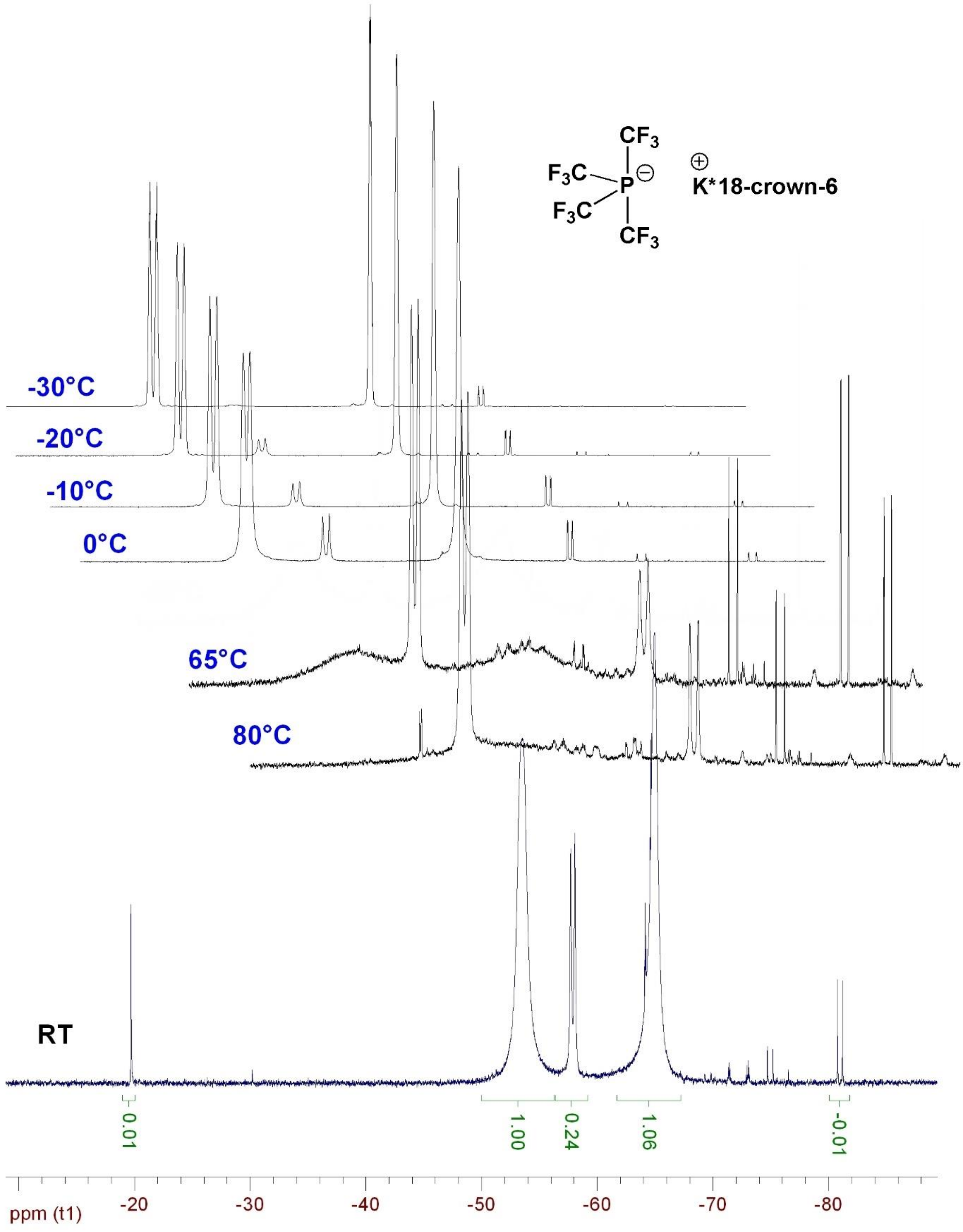

*Fig. S7: Temperature dependent ^{19}F NMR spectra of [K(18-crown-6)][P(CF_3)$_4$] (**2**) in monoglyme.*

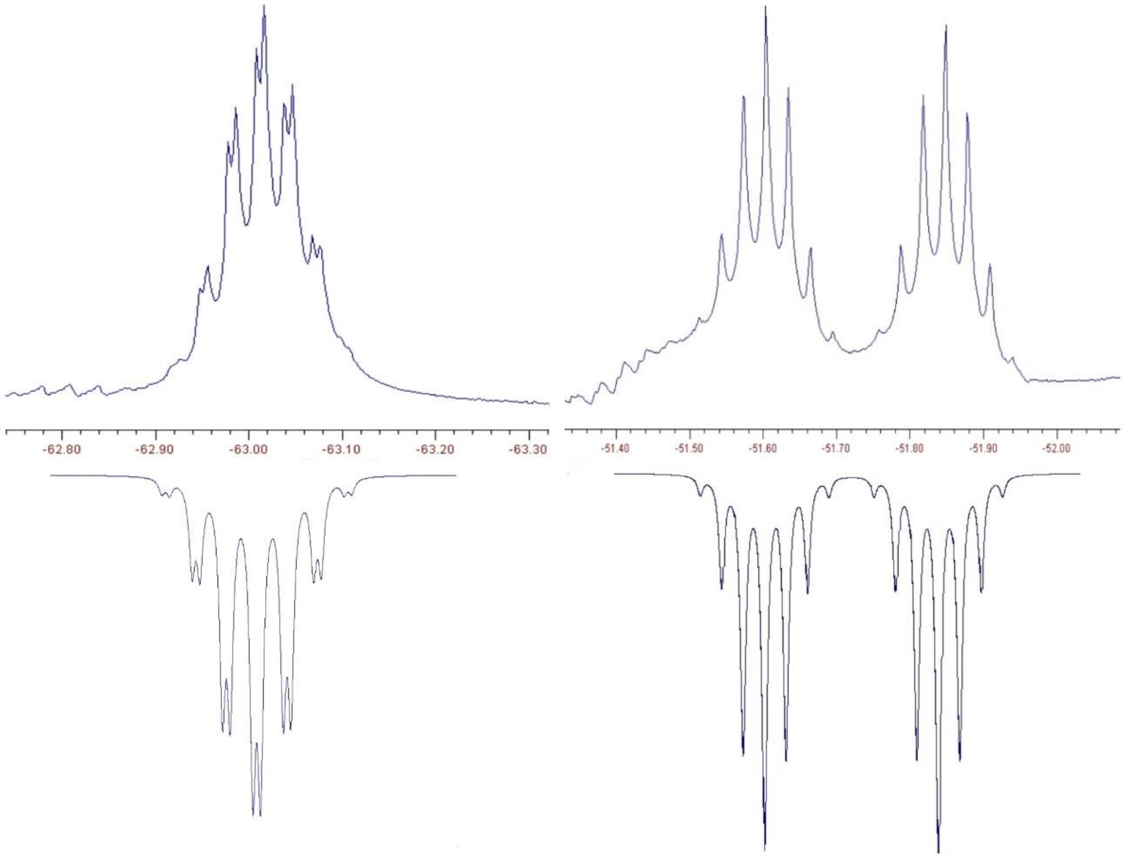

*Fig. S8: Experimental (top) and simulated (bottom) NMR spectra of [K(18-crown-6)][P(CF_3)$_4$] (**2**) at −50 °C.*

5. References

[1] R. Schmutzler, L. Heuer, D. Schomburg, *Phosphorus, Sulfur Silicon Relat. Elem.* **1993**, *83*, 149-156.

[2] J. Grobe, J. Hegge, *Synlett* **1995**, 641-642.

[3] A. A. Kolomeitsev, F. U. Seifert, G.-V. Röschenthaler, *J. Fluorine Chem.* **1995**, *71*, 47-49.

[4] a) K. J. Packer, *J. Chem. Soc.* **1963**, 960-966; b) M. Görg, G.-V. Röschenthaler, A. A. Kolomeitsev, *J. Fluorine Chem.* **1996**, *79*, 103-104.

[5] A. A. Pinkerton, R. G. Cavell, *Inorg. Chem.* **1971**, *10*, 2720-2723.

[6] G.-V. Röschenthaler, A. A. Kolomeitsev, unpublished results.

[7] C. P. Andrieux, L. Gelis, J. M. Saveant, *J. Am. Chem. Soc.* **1990**, *112*, 786-791.

[8] W. Heilemann, R. Mews, *Chem. Ber.* **1988**, 121, 461-463.

[9] a) N. Muller, D. T. Carr, *J. Phys. Chem.* **1963**, 67, 112-115; b) K. B. Wiberg, K. W. Zilm, *J. Org. Chem.* **2001**, *66*, 2809-2817.

[10] A. A. Kolomeitsev, V. N. Movchun, N. V. Kondratenko, Yu. L. Yagupolski, *Synthesis* **1990**, 12, 1151-1152.

[11] T. Matsuda, T. Harada, N. Nakajima, T. Itoh, K. Nakamura, *J. Org. Chem*. **2000**, 65, 157-163.

[12] A. A. Kolomeitsev, A. A. Kadyrov, J. Szczepkowska-Sztolcman, M. Milewska, H. Koroniak, G. Bissky, J. A. Barten, G.-V. Röschenthaler, *Tetrahedron Lett.* **2003**, *44*, 8273-8277.

[13] S. P. Kotun, J. D. O. Anderson, D. D. DesMarteau, *J. Org. Chem.* **1992**, *57*, 1124-1131.

[14] a) V. A. Petrov, *J. Fluorine Chem.* **1995**, 73, 17-19; b) C. G. Krespan, D. V. England, *J. Am. Chem. Soc.* **1981**, *103*, 5598-5599.

[15] P. H. M. Budzelaar, gNMR version 4.1, Cherwell Scientific, Oxford/UK **1998**.
